# Chronic inflammation as a driving factor for sarcopenia: an update on pathophysiology and future therapeutic targets

**DOI:** 10.3389/fphar.2026.1733798

**Published:** 2026-02-23

**Authors:** Zihan Liang, Lin Zhang

**Affiliations:** 1 West China School of Medcine, Sichuan University, Chengdu, China; 2 West China Hospital, General Practice Ward/International Medical Center Ward, General Practice Medical Center, Sichuan University, Chengdu, Sichuan, China

**Keywords:** aging, inflammation, inflammatory cytokines, sarcopenia, treatment

## Abstract

Sarcopenia is a syndrome characterized by an age-related progressive decline in skeletal muscle mass, strength, and function. It represents a significant public health concern because of its adverse impact on the quality of life and prognosis of older adults. Chronic low-grade inflammation contributes to the pathophysiology of sarcopenia through multiple pathways, including cellular senescence, immunosenescence, oxidative stress, mitochondrial dysfunction, hormonal alterations, and gut microbiota dysbiosis. To elucidate the role of chronic inflammation in the development of sarcopenia, we systematically searched PubMed and Web of Science databases using combinations of keywords such as “sarcopenia,” “chronic inflammation,” “inflammaging,” “cytokines” and “muscle atrophy,” which specifically addressed mechanistic pathways linking inflammation to muscle loss and emerging therapeutic targets. Moreover, obesity, a chronic inflammatory condition, is associated with sarcopenia, leading to sarcopenic obesity, which further exacerbates muscle loss and functional impairment. In terms of interventions, exercise, nutritional supplementation, and combined approaches have demonstrated efficacy in improving muscle mass and function, as well as conferring demonstrable anti-inflammatory benefits. In addition to conventional hormonal therapies, pharmacological strategies, particularly anti-inflammatory agents and treatments targeting inflammatory pathways, show considerable therapeutic promise. This review systematically examines the central role of chronic inflammation in the development and progression of sarcopenia, as well as its underlying mechanistic basis. It also elaborates on the roles of key inflammatory cytokines, such as C-reactive protein (CRP), interleukin-6 (IL-6), and tumor necrosis factor-α (TNF-α), in regulating muscle protein metabolic balance and their potential utility as biomarkers. A deeper understanding of the relationship between inflammation and sarcopenia will not only help elucidate its complex pathogenesis but also offer critical directions for the future development of early diagnostic tools and targeted anti-inflammatory interventions.

## 1 Introduction

Sarcopenia, an age-related geriatric syndrome, has emerged as one of the major threats to the health and wellbeing of elderly individuals. It was officially classified as an independent disease entity by the World Health Organization (WHO) and assigned the code ICD-10-CM (M62.84) in 2016 (Cao and Morley, 2016).

Epidemiological studies report that the prevalence of sarcopenia varies widely, ranging from 8.55% to 36.5%, depending on ethnicity and diagnostic criteria. Among individuals aged 65–70 years, the prevalence of sarcopenia is 13%–24%, whereas it exceeds 50% in those over 80 years of age (Chen Z. et al., 2021; Petermann-Rocha et al., 2022; Yuan and Larsson, 2023). Owing to its high prevalence, sarcopenia represents a significant public health challenge and imposes a considerable economic burden on aging societies.

Sarcopenia was first introduced as a concept by Rosenberg (1997). The currently accepted definition originates from the 2018 consensus of the European Working Group on Sarcopenia in Older People (EWGSOP). This definition encompasses not only reduced muscle mass and strength but also impaired muscle function, characterizing sarcopenia as a progressive and generalized skeletal muscle disorder (Cruz-Jentoft et al., 2019). It is associated with a range of adverse outcomes, such as physical disability, frailty, falls, hospitalization, loss of independence, and increased mortality (Chen et al., 2020). Moreover, sarcopenia represents an independent risk factor for poor prognosis in numerous patients with solid tumors (Bossi et al., 2021).

**FIGURE 1:**
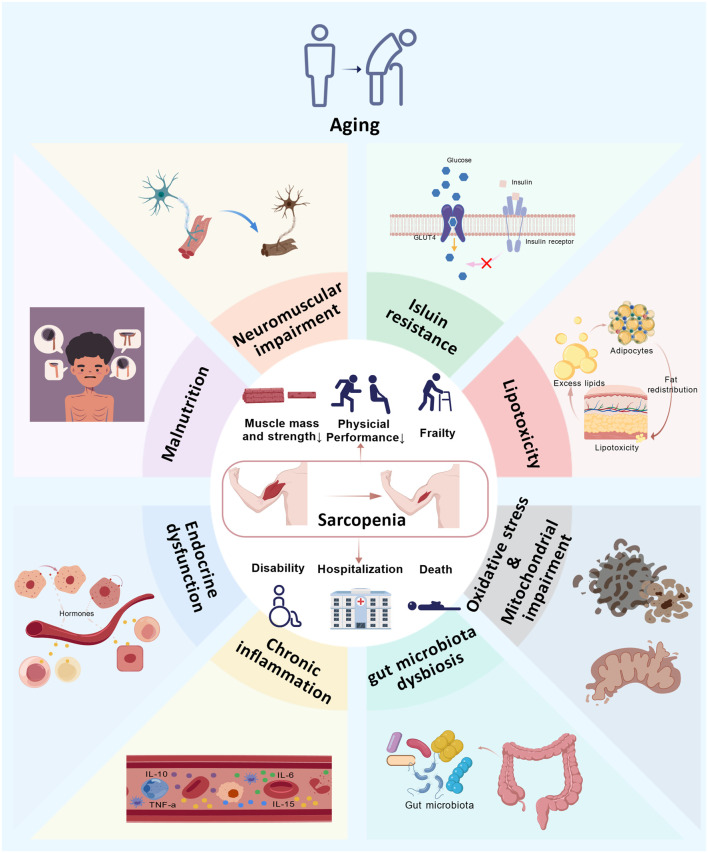
Multifactorial Mechanism of Sarcopenia. Aging, malnutrition, neuromuscular injury, insulin resistance, lipotoxicity, endocrine dysfunction, chronic inflammation, gut microbiota dysbiosis, oxidative stress, and mitochondrial dysfunction are all potential mechanisms underlying sarcopenia. Aging may further alter these pathological processes, and these processes may also interact with one another. Individuals with sarcopenia may experience adverse outcomes including reduced muscle mass and strength, decreased physical activity, frailty, disability, hospitalization, and even mortality.

The pathogenesis of sarcopenia is multifactorial and involves several interrelated mechanisms, including aging, malnutrition, neuromuscular impairment, insulin resistance, lipotoxicity, endocrine dysfunction, oxidative stress, mitochondrial impairment, and chronic inflammation (Figure 1). Although many underlying processes remain incompletely understood, they ultimately converge on an imbalance between muscle protein synthesis (MPS) and muscle protein breakdown (MPB) (Xia et al., 2017). Growing evidence indicates that chronic inflammation disrupts this balance by activating multiple molecular pathways that promote MPB (Bennett et al., 2023). Elevated levels of circulating inflammatory cytokines such as tumor necrosis factor-α (TNF-α), interleukin-6 (IL-6), C-reactive protein (CRP) and various chemokines have been consistently observed in sarcopenic individuals. These mediators are thought to exacerbate muscle loss through the activation of signaling cascades such as the nuclear factor-κB (NF-κB) pathway, which promotes inflammatory cell infiltration into muscle tissue (Zhang et al., 2022). Obesity, characterized by systemic low-grade chronic inflammation, also plays a significant role. The adipose tissue of obese individuals shows substantial immune cell infiltration and the secretion of inflammatory cytokines, including IL-6 and TNF-α (Kunz et al., 2021), thereby contributing to an inflammatory milieu that may accelerate sarcopenia (Wei et al., 2023). In summary, these findings underscore a strong association between inflammation and sarcopenia.

Chronic inflammation arises from persistent physical, chemical, or metabolic stimuli, often termed damage-associated molecular patterns (DAMPs),which continuously activate inflammatory signaling (Baechle et al., 2023). When the body cannot adequately resolve these responses, a chronic, low-grade, non-infectious state ensues. Unlike acute inflammation, this condition may last for months or years, frequently accompanied by tissue injury or dysfunction, and is closely linked to chronic diseases such as cardiovascular disorders, diabetes, and arthritis. Notably, in aging individuals, this low-grade inflammatory state in the absence of overt infection is described as inflammaging, a concept first introduced by Franceschi et al. (2000). Inflammaging extends from immunosenescence and is characterized by chronic, excessive activation of the innate immune system, coupled with a decline in adaptive immunity that impairs the clearance of senescent cells and inflammatory mediators. Consequently, pro-inflammatory markers remain elevated in cells and tissues. The gradual accumulation of this low-grade inflammation further accelerates aging, establishing a vicious cycle. Thus, inflammaging represents a distinct form of chronic inflammation in the aging organism and serves as a critical link between aging and age-related diseases, including sarcopenia. In the following sections, we will focus on the pivotal roles of chronic inflammation and inflammaging in the pathogenesis of sarcopenia.

This review aims to elucidate the dynamic interplay between chronic inflammation and sarcopenia, with a focus on the roles of inflammaging and obesity-related inflammation and inflammatory cytokines in its development and progression. It also summarizes current interventions for sarcopenia (Table 1), discusses the potential and challenges of anti-inflammatory strategies, and suggests directions for future research.

**TABLE 1:** Summary of different interventions for sarcopenia.

Intervention type	Specific measures	Primary mechanism	Intervention evaluation
Exercise intervention	Resistance training	Induce neuromuscular adaptations Modulate inflammation via myokine/adipokine regulation Inhibition of NF-κB/NLRP3 pathways	First-line intervention Improve muscle mass, strength, gait speed Reduce CRP, IL-6 Anti-inflammatory effects
	Aerobic exercise	Reduce systemic proinflammatory cytokines Improve mitochondrial function and oxidative stress	Lower inflammatory markers Iimprove exercise tolerance and supports resistance training adaptation
Nutritional intervention	Adequate protein	Activate mTOR signaling, stimulating myoplasmic/myofibrillar protein synthesis	Mitigate age-related muscle loss Improve muscle mass and function Enhanced effects when combined with exercise
	Vitamin D	Promote muscle cell proliferation/differentiation Reduce expression of atrophy-related proteins Inhibit NF-κB pathway	Improve muscle mass in deficient patients Evidence on strength improvement remains inconsistent
	Polyunsaturated fatty acids	Exert anti-inflammatory and immunomodulatory effects Activate mTOR Improve insulin sensitivity Reduce ROS	Reduce IL-6, IL-1β, TNF-α, CRP Improve protein synthesis, muscle mass, and function
	Low DII score diet	Reduce systemic inflammation	Anti-inflammatory diets may prevent/delay sarcopenia
Combined exercise and nutrition intervention		Synergistic effects on muscle anabolism and inflammation reduction	Superior to single-modality approaches Consistently improve body composition, physical function Reduce inflammatory markers
Traditional medicine	Testosterone	Increase lean mass, reduce fat mass in hypogonadal men	Increase muscle mass Effect on strength/performance limited Safety concerns
	Selective androgen receptor modulators	Anabolic effects on bone/muscle without stimulating non-skeletal tissues	Improve muscle mass and function Long-term safety and clinical efficacy in elderly unclear
Anti-inflammatory drugs	Nonsteroidal anti-inflammatory drugs	Systemic reduction of inflammation	Regular use associated with lower sarcopenia risk May preserve muscle mass/function
	TNF-α inhibitor	Block TNF-α activity, preventing muscle atrophy and fiber degradation	Prevent type II fiber atrophy in models Improve muscle strength/volume
	IL-6 receptor antagonist	Inhibit IL-6 signaling, reduces inflammation	Increase lean body mass
	Targeted therapy of inflammatory pathways	Inhibit key pathways involved in inflammation and muscle atrophy	Prevent muscle wasting Reduce systemic inflammation Improve muscle functio Clinical translation to primary sarcopenia remains challenging

## 2 Age-related inflammation (inflammaging) and sarcopenia

### 2.1 Cellular senescence-related inflammation and sarcopenia

Cell senescence is a stable cellular response first identified by Leonard Hayflick and Paul Moorhead in the 1960s (Hayflick and Moorhead, 1961). It refers to a stable state in which cells undergo irreversible cell cycle arrest after a finite number of divisions in response to various stressors, accompanied by a distinct secretory phenotype (Huang et al., 2022). Consequently, it is considered a fundamental mechanism of aging and a contributor to multiple age-related diseases. Cell senescence is a double-edged sword. While it protects against cancer by halting the proliferation of damaged cells, the accumulation of senescent cells can impair tissue homeostasis and promote age-related pathologies such as sarcopenia (Calcinotto et al., 2019). Cell senescence influences the development and progression of sarcopenia both directly through intrinsic muscle pathways and indirectly via senescence-associated inflammation. Elucidating the role of senescence in sarcopenia may offer novel perspectives and strategies for its intervention.

During cellular senescence, many *in vitro* and *in vivo* experimental models have revealed that senescent cells can not only alter the self-renewal capacity of myoblasts and the microenvironment within skeletal muscle cells by upregulating several antiapoptotic pathways, such as the p53/p21Cip1 pathway, the cyclin-dependent kinase inhibitor p16INK4A protein/pRB pathway, and the PI3K/Akt pathway (Mankhong et al., 2020; Suryadevara et al., 2024),but also directly diminish muscle myogenic differentiation potential. Additionally, senescent cells enhance inflammatory responses by secreting many proinflammatory factors and chemokines through the senescence-associated secretory phenotype (SASP). These two mechanisms jointly mediate the onset and progression of sarcopenia (Birch and Gil, 2020).

Senescent cells produce a distinct secretory profile known as the SASP, accompanied by transcriptional, epigenetic, morphological, and metabolic alterations that modify the tissue microenvironment and influence neighboring cells. The SASP encompasses a diverse array of signaling molecules, including proteases, coagulation factors, ceramides, bradykinins, extracellular matrix components, and damage-associated molecular patterns (DAMPs). Its composition is highly heterogeneous and varies depending on the cell type and the specific trigger of senescence (Birch and Gil, 2020). Evidence suggests that certain proteins may be consistently present across different forms of SASP and could modulate paracrine signaling through pathways such as MMP2–TIMP2 and IGFBP3–PAI-1 (SERPINE1) (Özcan et al., 2016). The regulation of SASP gene expression involves multiple pathways, with the p38 mitogen activated protein kinase (p38MAPK)/NF-κB axis recognized as a central driver. The activation of p38MAPK enhances the mRNA stability and transcription of SASP components, partly through NF-κB signaling. Consistent with these findings, inhibition of p38MAPK via SB203580 significantly reduces SASP production (Grun et al., 2023).

Senescent cells in skeletal muscle include fibroblasts, osteocytes, and endothelial cells. However, every cell type within the body is susceptible to senescence, leading to the secretion of a SASP rich in soluble mediators such as IL-1, IL-6, IL-8, IL-13, IL-18, TNF, and their receptors, collectively fostering an inflammatory microenvironment. These cytokines have been identified as contributors to sarcopenia susceptibility (Ferrucci and Fabbri, 2018; Hong et al., 2022), indicating that SASP accumulation can trigger chronic, low-grade inflammation that may be closely linked to muscle wasting.

Furthermore, studies indicate that SASP-driven inflammaging impairs muscle stem cell (MuSC) function. SASP components recruit and activate immune cells, which interact with proteases released by senescent cells, potentially promoting extracellular matrix (ECM) deposition. This process thickens the basement membrane surrounding MuSCs and compromises their regenerative capacity (Gopinath and Rando, 2008). Other evidence suggests that inflammation can reduce MuSC numbers, possibly through cytokine-mediated activation of the ubiquitin–proteasome system (Pascual-Fernández et al., 2020), which accelerates myosin degradation and muscle fiber loss. This may represent a plausible mechanism of sarcopenia development. Additionally, a big data model has shown that cells exhibiting the SASP promote the senescence of neighboring cells, thereby accelerating cellular aging (Katzir et al., 2021). Senescent cells also resist apoptosis and inhibit autophagy through altered signaling pathways and gene expression. For example, senescent fibroblasts overexpress BCL-2 to evade apoptosis, even when it is potentially triggered by their own SASP(He et al., 2021).

In summary, senescent cells foster an inflammatory niche through potent paracrine signaling and alter the behavior of nearby cells, thereby contributing to the pathophysiology of sarcopenia.

### 2.2 Immune aging-related inflammation and sarcopenia

Immune system aging encompasses immunosenescence and inflammaging. Immunosenescence involves the structural involution of immune organs and a functional decline in immune responsiveness, characterized by thymic atrophy and T-cell dysfunction affecting both innate and adaptive immunity (Thomas et al., 2020). The close association between immune aging and inflammation stems from inflammaging caused by impaired clearance of senescent somatic cells (SSCs). This environment weakens adaptive immunity, diminishes defenses against pathogens and the response to vaccination, and contributes to the development of multiple age-related diseases (Santoro et al., 2021; Fulop et al., 2023). For many years, immune aging has been regarded as detrimental. However, this perspective has been revised. The current understanding proposes that it is not merely harmful to the human body but rather a combination of poor adaptability/resilience and impaired adaptive capacity of the immune system, as it changes with age and is closely linked to immunobiography (Fulop et al., 2017).

Individual variations in inflammatory regulation significantly shape the trajectory of healthy aging (Santoro et al., 2021). A robust anti-inflammatory capacity promotes longevity and delays the onset of age-related diseases, whereas a predominance of inflammatory responses in older adults increases susceptibility to chronic conditions (Saini et al., 2016). A close association exists between sarcopenia and immune aging, which is supported by accumulating evidence. Animal models suggest that immunosenescence may disrupt skeletal muscle protein metabolism by blunting the postprandial rise in muscle protein synthesis and suppressing proteolysis (Palla et al., 2021). Moreover, immune dysregulation due to immune aging has been linked to impaired exercise tolerance, potentially accelerating the progression of sarcopenia (Bhanji et al., 2019).

Originally thought to be unaffected by aging, innate immunity is now recognized as undergoing significant functional decline with age. In the skeletal muscle of older adults, this decline manifests as impaired activity of neutrophils, macrophages, and dendritic cells. This aging process alters the immune microenvironment of skeletal muscle and contributes to the development of sarcopenia (Zhang et al., 2022). Neutrophils exhibit reduced phagocytosis, degranulation, and reactive oxygen species production, diminishing their bactericidal capacity and amplifying inflammation (Zhang et al., 2024). Elevated neutrophil numbers are considered a compensatory response to this functional impairment. The neutrophil-to-lymphocyte ratio has been established as a prognostic marker in sarcopenia, with higher neutrophil counts and lower lymphocyte counts correlating with reduced muscle strength (Angulo et al., 2016). In addition to promoting inflammation, neutrophils can directly damage muscle cell membranes via superoxide-dependent mechanisms (Xiang et al., 2021) and impair muscle repair through deficient recruitment, both of which may accelerate sarcopenia. Macrophage aging is characterized by diminished phagocytic ability and a shift toward the anti-inflammatory M2 phenotype. However, premature or excessive conversion to the anti-inflammatory phenotype can also impair muscle health (Wang et al., 2024). Animal models and human tissue studies have demonstrated that aging muscle tissue exhibits a significant increase in the number of anti-inflammatory macrophage phenotypes, including the CD206+ phenotype, which negatively impacts skeletal muscle mass (Reidy et al., 2018; Wang et al., 2019; Brorson et al., 2025). Similarly, during aging, dendritic cells exhibit increased release of proinflammatory cytokines alongside reduced numbers of circulating cells, decreased migratory capacity, and diminished phagocytic function. Consequently, this process of immune senescence may foster an inflammatory environment that disrupts skeletal muscle regeneration and promotes atrophy (Liu J. et al., 2021).

Adaptive immune aging involves a functional decline in T and B lymphocytes, with T-cell alterations being particularly relevant to the development of sarcopenia. T lymphocytes originate as immature cells in the thymus and mature in peripheral lymph nodes. In aged skeletal muscle, the number of naive T cells decreases, T-cell receptor (TCR) diversity decreases, and memory T-cell populations expand, impairing responses to novel antigens (Shin et al., 2019). During the process of immune aging, the reduction and shift of T cells from the CD8^+^ phenotype to the CD4^+^ phenotype may be associated with muscle mass loss (Huang et al., 2020). Furthermore, T cells secrete cytotoxic granules and proinflammatory cytokines that sustain a potent inflammatory response. Elevated TNF-α, for example, promotes muscle atrophy and weakness through multiple pathways and enhances the accumulation of CD28-null T cells, forming a vicious cycle that exacerbates sarcopenia (Wang et al., 2018). Regulatory T cells (Tregs), which infiltrate skeletal muscle, help modulate local immunity by inducing neutrophil apoptosis and secreting anti-inflammatory cytokines such as IL-10 and transforming growth factor-β (TGF-β) to influence macrophage behavior. However, Treg depletion during immune aging amplifies Interferon-gamma (IFN-γ) responses, leading to aberrant muscle inflammation, fibrosis of regenerating fibers, and eventual muscle loss (Xiang et al., 2021). B-cell function also decreases with immune senescence (Dowery et al., 2021), potentially hindering muscle regeneration and compromising strength recovery. Nevertheless, the exact mechanisms underlying B-cell involvement in sarcopenia require further elucidation.

In summary, alterations in the function and number of various immune cells within the aging immune system impact skeletal muscle regenerative potential, muscle mass, and strength through multiple mechanisms. Furthermore, the abnormal inflammatory responses triggered by immune senescence contribute to the onset and progression of sarcopenia.

### 2.3 Oxidative stress- and mitochondrial dysfunction-related inflammation and sarcopenia

Oxidative stress refers to a state in which the dynamic equilibrium between reactive oxygen species (ROS) production and the body’s antioxidant defense mechanisms is disrupted, leading to excessive accumulation of ROS. This imbalance favors an oxidized state, causing elevated intracellular ROS levels that damage vital biomolecules and cells, affecting the entire organism. It is considered a major factor contributing to aging and disease (Xu et al., 2021).

ROS function as signaling molecules that activate defense mechanisms and initiate inflammatory responses. Under physiological conditions, ROS are maintained at low levels through tightly regulated antioxidant systems. In aging, however, diminished antioxidant capacity disrupts the redox balance, leading to the accumulation of free radicals and the induction of systemic inflammation. Substantial evidence indicates that ROS activate Toll-like receptors on various immune cells, playing a key role in triggering inflammatory cascades (Li and Chang, 2021). Concurrently, ROS act as important second messengers in immune signaling. Elevated ROS levels in immune cells, which serve as secondary mediators of cytokines such as TNF-α in skeletal muscle, directly or indirectly activate the NF-κB pathway, promoting tissue damage and potentially contributing to the pathogenesis of sarcopenia. Studies have shown that aging elevates basal oxidative stress in skeletal muscle, which is further amplified during disuse atrophy, suggesting a role for oxidative stress in both inactivity-related muscle loss and sarcopenia (Zhang et al., 2023). In a cross-sectional clinical study of 140 elderly patients with mild to moderate Alzheimer’s disease (AD), the levels of oxidative stress markers such as 8-hydroxy-2′-deoxyguanosine (8-OHdG) and 8-isoprostane were significantly correlated with frailty severity, indicating the involvement of oxidative stress in the pathophysiology of frailty in AD (Namioka et al., 2017). Research in animal models has confirmed that oxidative stress leads to age-related muscle fiber damage and loss (Tang et al., 2019).

Mitochondria-derived adenosine triphosphate (ATP) serves as the primary energy carrier for skeletal muscle contraction, regulating muscle maintenance and function. Consequently, both mitochondrial integrity and muscle homeostasis rely on tightly controlled mitochondrial protein quality (Hood et al., 2019). Studies have shown that the accumulation of damaged and misfolded mitochondrial proteins increases with age, disrupting mitochondrial homeostasis. This disturbance elevates ROS production, which promotes inflammatory activation within skeletal muscle tissue (Picca et al., 2017; Leduc-Gaudet et al., 2021). As the major source of ROS in muscle, mitochondria are highly vulnerable to oxidative damage, particularly damage to mitochondrial DNA (mtDNA), due to their proximity to the electron transport chain (Peoples et al., 2019). Mitochondrial injury releases DAMPs, such as acyl peptides and mtDNA, initiating a self-sustaining cycle of dysfunction and oxidative stress (Marchi et al., 2023). Furthermore, mitochondrial DAMPs (MTDs) activate the NLRP3 inflammasome, leading to caspase-1 activation and subsequent secretion of IL-1β and IL-18 (Xian et al., 2022), enhancing mitochondrial ROS generation and promoting inflammatory tissue injury.

Overall, the self-reinforcing cycle of oxidative stress, disrupted mitochondrial proteostasis, and inflammaing forms a critical link between aging and sarcopenia. Interventions targeting oxidative damage and mitochondrial dysfunction may thus represent promising therapeutic strategies for sarcopenia.

### 2.4 Others

Age-related inflammation contributes to sarcopenia progression through multiple pathways. In addition to the aforementioned pathways, endocrine alterations associated with aging and dysbiosis of the gut microbiota in older individuals also play a significant role. During aging, declining growth hormone levels promote adipose accumulation and muscle loss. Similarly, reduced secretion of insulin-like growth factor-1 (IGF-1) affects the expression of genes involved in inflammation and autophagy (Clegg and Hassan-Smith, 2018; Bartke, 2021). The hypothalamic‒pituitary‒adrenal (HPA) axis becomes markedly activated with age, leading to elevated glucocorticoid secretion, which further suppresses growth hormone release and may impair IGF-I activity in peripheral tissues (Lopes et al., 2019; Bian et al., 2020). Testosterone is well established for its positive effects on muscle mass and strength. The age-related decline in testosterone levels is closely associated with sarcopenia development (Shin et al., 2018; Kraemer et al., 2020). Similarly, dehydroepiandrosterone (DHEA) secretion gradually decreases with age, and lower DHEA levels are correlated with elevated IL-6, but its clinical significance requires further clarification (James et al., 1997).

The gut microbiota maintains systemic connections with multiple organs and tissues, playing a vital role in immune and metabolic regulation (Franceschi et al., 2018). Substantial evidence supports a strong link between age-related gut microbial changes and inflammaing (Clemente et al., 2018). With aging, increased intestinal mucosal permeability and impaired barrier function facilitate the translocation of bacteria and their metabolites into the bloodstream, triggering systemic inflammation and increasing the levels of proinflammatory cytokines such as TNF-α and IL-6 (Grosicki et al., 2018). This age-related inflammation and microbial dysbiosis may further exacerbate intestinal barrier dysfunction, creating a vicious cycle of bacterial translocation and inflammatory activation (Thevaranjan et al., 2017). The “gut‒muscle axis” hypothesis provides a more comprehensive perspective and explanation linking gut dysbiosis, inflammation, and sarcopenia. Through this complex network, gut-derived hormones and metabolites interact with skeletal muscle, disrupting energy metabolism and promoting protein breakdown. This process amplifies the release of inflammatory mediators, initiating local and systemic inflammatory responses that ultimately reduce muscle mass and strength (Ticinesi et al., 2017; de Sire et al., 2018). Aging-associated gut microbial metabolites can also directly impair muscle regeneration. For example, indole-3-sulfate (IS) promotes muscle atrophy by enhancing inflammation, inducing excessive antioxidant responses, and impairing mitochondrial function. Similarly, sulfuric acid and p-cresol derived from putrefactive bacteria may contribute to insulin resistance and increased intramuscular lipid deposition, worsening muscle quality (Liu C. et al., 2021).

In summary, age-related inflammation involves multiple interacting systems and pathways, which also influence the onset and progression of sarcopenia through various complex mechanisms. However, the specific pathways of inflammation and the precise role of inflammatory mediators in sarcopenia remain incompletely understood. Further basic and clinical studies, particularly prospective investigations, are needed to clarify these mechanisms.

## 3 Obesity-related inflammation and sarcopenia

Obesity, characterized by excessive adipose tissue accumulation, has become a worldwide epidemic that significantly increases the risk of type 2 diabetes, cardiovascular disease, nonalcoholic fatty liver disease, and certain cancers. Adipose tissue functions not only as an energy storage depot but also as an active endocrine organ. It secretes various adipokines, including adiponectin and leptin (Kalinkovich and Livshits, 2017), that help regulate food intake, energy balance, insulin sensitivity, thermogenesis, and immune function (Sakers et al., 2022). In obesity, adipocyte hypertrophy and hyperplasia drive the secretion of proinflammatory cytokines such as TNF-α and IL-6, leading to a state of low-grade chronic inflammation. This condition promotes insulin resistance and lipid accumulation, particularly in elderly individuals (Kahn et al., 2019; Kojta et al., 2020; Reyes-Farias et al., 2021). Consequently, obesity is recognized both as a manifestation of systemic low-grade inflammation and as a key contributor to the pathogenesis of sarcopenia (Li et al., 2022).

### 3.1 Adipose tissue and inflammation

Adipose tissue is broadly categorized into subcutaneous (SAT) and visceral (VAT) depots. Under conditions such as high dietary fat intake, limited SAT expandability, impaired lipid metabolism, or aging, lipids may aberrantly accumulate in normally low-fat organs, including skeletal muscle, a process termed ectopic fat deposition (Girousse et al., 2019). This ectopic lipid infiltration disrupts normal tissue and organ function. Within skeletal muscle, ectopic fat manifests as intramyocellular lipids (IMCLs) and intermuscular adipose tissue (IMATs). This deposition may result from increased adipocyte lipolysis, leading to excessive release of free fatty acids (FFAs) into the circulation (Yoon et al., 2021). Ectopic lipids, particularly IMAT, are recognized as key contributors to insulin resistance and local inflammation (Cheng et al., 2023). IMAT promotes the secretion of inflammatory cytokines such as TNF-α and IL-6 within muscle, which in turn impairs muscle mass, strength, and function (Stout et al., 2017; Goodpaster et al., 2023). A clinical study comparing different patient subgroups revealed that the sarcopenic obesity and obesity groups presented the highest IMAT levels. Elevated IMAT was significantly correlated with increased levels of the inflammatory marker chemoattractant protein-1 (MCP-1), as well as reduced walking speed and muscle strength, suggesting a link between IMAT-driven inflammation and functional decline. In parallel, IMCL accumulation can disrupt mitochondrial function by reducing fatty acid β-oxidation and increasing ROS production, creating a lipotoxic intramuscular environment that further promotes lipid accumulation and insulin resistance (JafariNasabian et al., 2017; Kalinkovich and Livshits, 2017). Additionally, IMCLs attract immune cells such as macrophages, T cells, and B cells into muscle tissue, where they secrete proinflammatory factors and chemokines that exacerbate local inflammation (Kalinkovich and Livshits, 2017).

In addition to local inflammation in tissues such as skeletal muscle, adipose tissue significantly contributes to systemic chronic inflammation. With the development of obesity, macrophages polarize toward a proinflammatory phenotype, resulting in the accumulation of M1-type macrophages that secrete substantial amounts of proinflammatory cytokines, including TNF-α, IL-6, and IL-1β, thereby sustaining systemic low-grade chronic inflammation (Mouton et al., 2020). Crosstalk between macrophages and adipose tissue further amplifies this process. In obesity, hypertrophic adipocytes secrete chemokines, such as monocyte MCP-1, which promote the infiltration of monocytes and macrophages into adipose depots. These macrophages, in turn, release proinflammatory mediators such as TNF-α, which increases MCP-1 promoter activity in adipocytes. Concurrently, excessive FFAs released by adipocytes activate macrophages, whose subsequent secretion of TNF-α feeds back to adipocytes, thereby enhancing inflammatory responses and stimulating further FFA release. This positive feedback loop intensifies inflammatory signaling, establishing a self-perpetuating cycle that exacerbates adipose tissue inflammation (Engin, 2017).

As a secretory organ, adipose tissue releases adipokines such as leptin and adiponectin, which appear to influence sarcopenia through inflammatory pathways (Bilski et al., 2022). Leptin exerts proinflammatory effects, partly by increasing the production of inflammatory cytokines such as TNF-α, IL-6, and IL-12 in monocytes (Pérez-Pérez et al., 2020). A cross-sectional study demonstrated that plasma leptin levels were significantly and independently negatively correlated with thigh muscle cross-sectional area (CSA) in middle-aged and older adults. Notably, leptin concentrations were markedly elevated in individuals with sarcopenic visceral obesity (5.7 μg/L in men) compared to those with visceral obesity alone (4.3 μg/L) or no sarcopenia/obesity (1.9 μg/L), suggesting a link between leptin, visceral fat, and muscle mass (Kohara et al., 2011). The mechanism of leptin’s action on muscle remains unclear. While aging rodent models involve elevated leptin with increased IMAT and muscle atrophy, leptin treatment in leptin-deficient (ob/ob) mice increases muscle mass and downregulates atrophy-related genes, including myostatin, muscle RING-finger protein-1 (MuRF1), and atrogin-1 (Sáinz et al., 2009). In contrast to leptin levels, adiponectin levels inversely correlate with fat mass and decrease with age and obesity (Voss et al., 2016). Adiponectin acts as a positive regulator of muscle function with anti-inflammatory properties (Gianopoulos et al., 2025); it promotes fatty acid oxidation, improves insulin sensitivity, and inhibits macrophage polarization to the proinflammatory M1 phenotype while stimulating anti-inflammatory M2 activation, thereby reducing the levels of proinflammatory cytokines and increasing the levels of anti-inflammatory mediators such as IL-10 (Luo and Liu, 2016; Choi et al., 2020). Chinese cross-sectional, cohort, and intervention studies reported that elevated inflammatory markers such as TWEAK (>1276.48 pg/mL) and TNF-α (29.11 ± 22.67 pg/mL) increased sarcopenia risk, whereas metabolic factors such as IGF-1 (62.13 ± 22.52 ng/mL), insulin (22.19 ± 17.36 pmol/L), and adiponectin (median 3.53 μg/mL) were protective (Li et al., 2019). However, similar to the dual role of leptin, increased circulating adiponectin has also been associated with reduced muscle strength, impaired physical function, and an increased prevalence of frailty and sarcopenia in several studies (Huang et al., 2015; Adachi et al., 2020; Komici et al., 2021).

Overall, muscle factors such as leptin and adiponectin are associated with the development of sarcopenia. However, given that most studies are observational, their precise roles and the mechanisms through which they influence sarcopenia remain unclear. Further long-term prospective studies are needed to explore these questions.

### 3.2 Aging, adipose tissue, and inflammation

A complex interplay exists among aging, obesity, and inflammation. Studies indicate that adipose tissue undergoes aging early in the lifespan and represents one of the most vulnerable tissues in the aging process (Ou et al., 2022). Understanding the mechanisms connecting aging, obesity, and inflammation may offer valuable insights into the pathogenesis of sarcopenia and reveal potential intervention targets.

During aging, adipocytes frequently undergo hypertrophy while their number decreases, accompanied by morphological and functional alterations. Age-related dysregulation of lipid metabolism contributes to fat redistribution, often leading to increased visceral adiposity and obesity (Nguyen and Corvera, 2024). The differentiation and proliferative capacity of adipose-derived stromal cells (ASCs) are essential for maintaining functional adipose tissue (Bunnell, 2021). With aging, however, ASC function decreases, impairing tissue renewal and expansion while reducing metabolic plasticity, a decline that may contribute to insulin resistance in older adults (Murakami et al., 2022). Impaired differentiation and lipid storage in preadipocytes also lead to increased systemic exposure to FFAs and lipotoxicity (Ou et al., 2022). Notably, immune cells and adipose-derived stromal cells, rather than mature adipocytes, appear to be the primary sources of inflammatory mediators in age-related adipose tissue inflammation (Nguyen and Corvera, 2024). Under TNF-α stimulation, preadipocytes release proinflammatory cytokines that induce a proinflammatory state in neighboring cells, promote monocyte adhesion to endothelial cells, and recruit macrophages, thereby amplifying inflammation. During aging, preadipocytes may dedifferentiate into a proinflammatory, senescence-like state, which similarly triggers immune cell infiltration. These immune cells can further activate preadipocytes to sustain a proinflammatory feedback loop (Frasca and Blomberg, 2020). Additionally, senescent preadipocytes can induce the SASP (Chen X. et al., 2021). As noted previously, SASP factors recruit and activate macrophages, which release chemokines and inflammatory cytokines, thereby perpetuating chronic inflammation (Birch and Gil, 2020).

### 3.3 Vicious cycle of obesity and sarcopenia-sarcopenic obesity

**FIGURE 2:**
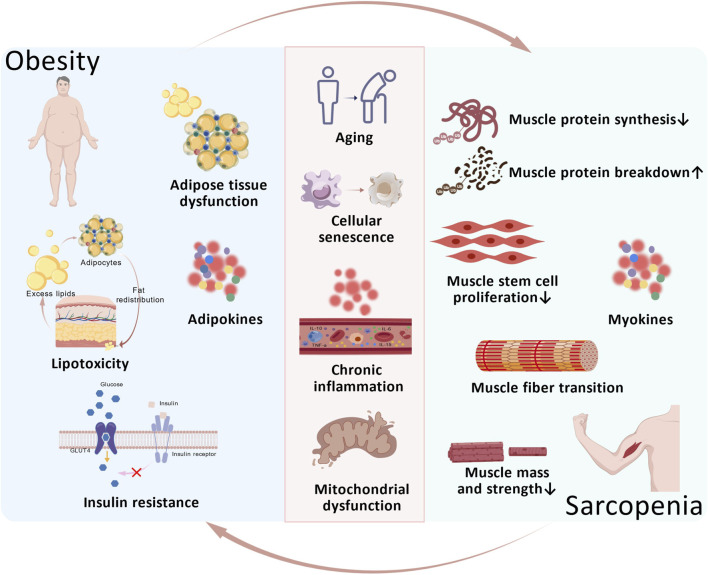
The Vicious Cycle of Obesity and Sarcopenia. With aging, obese individuals experience adipose tissue dysfunction which leads to a synergistic detrimental effect on skeletal muscle through localized and systemic inflammation, a lipotoxic environment, mitochondrial dysfunction, insulin resistance, and the secretion of adipokines. In sarcopenia patients, an imbalance between muscle protein synthesis and degradation leads to reduced muscle mass and quality, accompanied by decreased muscle stem cell proliferation and impaired mitochondrial function. Simultaneously, reduced patient activity levels and the impact of myokines on adipose tissue metabolism further promote fat accumulation, exacerbating obesity. When obesity and sarcopenia coexist, a vicious cycle emerges involving inflammation, aging, and interactions between adipose tissue and skeletal muscle, accelerating the progression of both conditions.

With advancing age, the accumulation of intramuscular and visceral adipose tissue increases, increasing the risk of obesity. The associated local and systemic inflammation, lipotoxic environment, mitochondrial dysfunction, and insulin resistance collectively exert detrimental effects on skeletal muscle. These changes increase susceptibility to sarcopenia and accelerate its progression. In sarcopenic individuals, reduced muscle mass and quality result from a disrupted balance between muscle protein synthesis and degradation, coupled with mitochondrial impairment. Concomitantly, diminished physical activity in these patients further promotes adipose accumulation, exacerbating the onset of obesity. Skeletal muscle itself functions as an endocrine organ, secreting myokines such as IL-6 and TNF-α that can influence adipose tissue metabolism. When obesity and sarcopenia coexist, the interplay among inflammation, aging, adipose tissue, and skeletal muscle forms a vicious cycle that accelerates both conditions (Figure 2).

This clinical presentation has been termed sarcopenic obesity (SO), which is defined by the concurrent presence of reduced muscle mass/function and excessive adiposity (Heber et al., 1996; Baumgartner, 2000). Although the concept of SO was introduced more than 2 decades ago, universally accepted diagnostic criteria remain elusive. Variations in age, sex, and ethnic characteristics affecting body composition have contributed to heterogeneous diagnostic approaches. Currently, the primary approach combines diagnostic criteria for sarcopenia and obesity (Donini et al., 2022).

The pathogenesis of SO involves multifaceted interactions among various factors. As previously described, aging, physical inactivity, poor nutrition, insulin resistance, chronic inflammation, and oxidative stress collectively contribute to the progressive loss of muscle mass and function, along with excessive fat accumulation. Muscle and adipose tissue share several inflammatory pathways and communicate via autocrine and paracrine signaling through secreted peptides. These common injury-related mechanisms create strong pathogenic links between the two tissues, enabling their mutual reinforcement in driving SO development (Lynch et al., 2020). Additional factors, such as fluctuations in sex hormones, including estrogen and testosterone, may further modulate muscle mass and fat distribution. Studies have shown that an overweight status impairs skeletal muscle autophagy (Potes et al., 2017), a process essential for maintaining insulin sensitivity and metabolic homeostasis. Dysregulated autophagy offers a molecular explanation for skeletal muscle atrophy (SMA) and represents a promising therapeutic target. Additionally, gut microbiota dysbiosis has been associated with impaired muscle function, systemic inflammation, and metabolic dysregulation, suggesting its potential role in SO (Prokopidis et al., 2020). As noted earlier, the gut‒muscle axis may participate in SO pathogenesis through the AMP-activated protein kinase (AMPK) and PGC-1alpha signaling pathways (Ticinesi et al., 2017). Whether this axis represents a viable target for SO intervention merits further investigation.

In the pathogenesis of SO, IR and chronic low-grade inflammation are two central and interrelated pathological drivers. IR, characterized by reduced insulin-mediated glucose uptake and compensatory hyperinsulinemia, is closely linked to the loss of skeletal muscle mass, a major insulin target tissue. This decline not only triggers IR but also impairs muscle metabolism, mitochondrial function, and protein synthesis, collectively contributing to SO. Debate persists regarding the primary initiating factor in SO. One perspective holds that IR serves as the core mechanism; through metabolic dysfunction, ectopic lipid accumulation, and oxidative stress, it promotes polarization of macrophages toward a pro-inflammatory M1 phenotype, thereby initiating chronic inflammation (Meiliana et al., 2024). Conversely, other evidence suggests that chronic low-grade inflammation—driven by visceral adipose expansion and dysfunctional adipose tissue in obesity—plays a key role in instigating and aggravating IR. Infiltrating adipose tissue macrophages release cytokines such as TNF-α and IL-6, which activate pathways including JNK and IKKβ/NF-κB, inhibit IRS-1 tyrosine phosphorylation, and suppress downstream PI3K/AKT signaling. Impaired insulin signaling attenuates lipolysis suppression, elevating circulating free fatty acids that in turn sustain immune activation via receptors such as TLRs, perpetuating a pro-inflammatory state. Although the sequence of IR and inflammation remains incompletely resolved, it is widely accepted that their mutual reinforcement forms a “metabaging cycle” (Li et al., 2022), a critical mechanism underlying the development and progression of sarcopenic obesity.

SO represents the convergence of two major public health challenges: population aging and obesity. However, not all obese older adults develop sarcopenia, indicating variability in individual susceptibility. In addition to individual differences, research suggests that excess weight can initiate an adaptive physiological response. Mechanical loading stimulates muscle and skeletal mechanoreceptors, promoting the synthesis of growth factors that facilitate muscle hypertrophy (Prado et al., 2024). In SO, however, this compensatory mechanism becomes unstable. While some individuals maintain proportionate muscle mass with weight gain, others exhibit adipose accumulation without corresponding increases in muscle mass or function (Batsis and Villareal, 2018). This imbalance in tissue remodeling likely contributes to SO development.

In summary, the causes of SO are complex, but the interplay among inflammation, aging, and muscle and fat tissues constitutes one of its key pathogenic mechanisms. Although the mechanistic understanding remains challenging, ongoing research continues to elucidate these complex relationships. Early identification and intervention for both sarcopenia and SO are essential for preserving functional capacity and quality of life in older adults.

## 4 Mechanisms of inflammatory cytokines in the development of sarcopenia

### 4.1 IL-6

IL-6, produced by nearly all stromal and immune cells, has pleiotropic effects. It not only regulates cell differentiation, proliferation, and apoptosis but also exerts hormone-like influences on vascular homeostasis, lipid metabolism, insulin sensitivity, mitochondrial function, and neuroendocrine signaling (Tanaka et al., 2018; Kaur et al., 2020; Kondo et al., 2021).

Skeletal muscle itself secretes IL-6, which is classified as a myokine that modulates muscle function and mass (Severinsen and Pedersen, 2020). During exercise, muscle-derived IL-6 promotes hypertrophy through satellite cell activation while enhancing fatty acid oxidation and glucose uptake (Belizário et al., 2016; Guo et al., 2017). This finding suggests that transient, contraction-induced IL-6 elevation supports muscle anabolism. However, chronically elevated IL-6, as observed in inflammatory states, induces muscle atrophy via ubiquitin‒proteasome activation, impairs insulin signaling, and suppresses anabolic pathways (Guo et al., 2017; Forcina et al., 2018). Pototschnig et al. demonstrated that mice implanted with IL-6-secreting fibrosarcoma cells developed cachexia, characterized by systemic inflammation, muscle atrophy, and weight loss, effects prevented by IL-6 knockout in cancer cells (Pototschnig et al., 2023). Clinical studies further support these conclusions. A cohort study of 690 elderly women revealed that elevated serum IL-6 (>3.10 pg/mL) levels were related to physical disability and decreased walking capacity (Ferrucci et al., 2002). A randomized trial involving 99 older adults revealed that age-related IL-6 elevation impaired muscle strength, mass, and adaptive response to training (Grosicki et al., 2020). A meta-analysis revealed an inverse correlation between plasma IL-6 and grip strength across sexes, although men with better muscle conditions presented higher IL-6 thresholds (mean difference: 0.25 pg/mL) than women did. These findings suggest that sex-specific reference values may be needed for the prediction of sarcopenia (Mikó et al., 2018). However, conflicting evidence exists. A meta-analysis of 3,072 sarcopenia patients and 8,177 controls revealed significantly elevated CRP (SMD = 0.51; 95%CI: 0.26, 0.77) but not IL-6 (SMD = 0.35; 95%CI: −0.19, 0.89) in sarcopenic individuals (Bano et al., 2017). Similarly, a cross-sectional study detected no correlation of IL-6 with muscle strength or physical performance (Liu H.-C. et al., 2021), potentially because of its small sample size (n = 77), diagnostic criteria relying solely on muscle mass, and confounding comorbidities. A meta-analysis of 168 studies confirmed negative correlations between IL-6, TNF-α, CRP, and muscle strength. Among these, CRP was more strongly associated with muscle mass (r = -0.12) than IL-6 (r = -0.09) and TNF-α (r = -0.15), especially in community-dwelling elderly individuals (Tuttle et al., 2020). The modest relationship between IL-6 and muscle strength may reflect its dependency on other inflammatory mediators to exert full catabolic effects (Belizário et al., 2016). The overall weak relationship between IL-6 and muscle strength may be due to its catabolic effects on muscle tissue, which require regulation by other proinflammatory cytokines. By acting alone, it is insufficient to induce the catabolic effects necessary for muscle wasting and atrophy.

In conclusion, while substantial evidence implicates IL-6 in muscle pathology, its utility as a standalone diagnostic or prognostic biomarker for sarcopenia requires further validation.

### 4.2 TNF-α

TNF-α, which is secreted by macrophages, monocytes, neutrophils, CD4^+^ T cells, and NK cells, functions as a key proinflammatory cytokine and central regulator of immune responses. It modulates tissue homeostasis by coordinating the production of other cytokines and regulating cell survival and death processes (Van Loo and Bertrand, 2023).

Upon receptor binding, TNF-α activates the MAPK and NF-κB signaling pathways (Ting and Bertrand, 2016), driving proinflammatory gene expression while reducing protein stability and impairing muscle stem cell proliferation and differentiation, ultimately leading to muscle atrophy (Wang et al., 2018). Wu et al. established a natural aging mouse model; elevated TNF-α was shown to activate the caspase-8/caspase-3 pathway, cleaving Gasdermin E (GSDME) and triggering pyroptosis, thereby directly promoting muscle fiber loss and functional decline (Wu et al., 2023). Consistent with these findings, Wang et al. reported that TNF-α knockout increased muscle stem cell numbers and reduced centrally nucleated myofibers, indicating that myeloid-derived TNF-α accelerates muscle aging by impairing regeneration and promoting aberrant fusion (Wang et al., 2018). TNF-α-deficient (TNFα-CKO) mouse models further demonstrated enhanced myoblast proliferation, migration, and differentiation and improved muscle regeneration, potentially through the upregulation of myogenic factors, altered protein localization, activation of oxidative phosphorylation, and suppression of Janus kinase-signal transducer and activator of transcription (JAK-STAT) signaling (Fu et al., 2024). Clinical evidence aligns with experimental data. A prospective study of 599 adults aged ≥85 years linked elevated innate TNF-α production with each doubling in LPS-stimulated production capacity, to a 1.3 kg accelerated decline in handgrip strength over 4 years (Taekema et al., 2007). Another study reported that high TNF-α and TWEAK levels increased sarcopenia risk by 7.6-fold and 14.3-fold, respectively (Li et al., 2019). However, a cross-sectional study of 299 participants revealed significantly lower TNF-α levels in older adults (≥65 years) with low muscle mass (7.0 ± 8.0 pg/mL) than in healthy controls (11.8 ± 11.6 pg/mL), with no significant differences observed in IL-6 and MCP-1 levels, suggesting that reduced muscle mass does not consistently correlate with elevated inflammatory markers (Ito et al., 2021). This discrepancy may stem from methodological differences, as current guidelines emphasize muscle strength, rather than mass, as the primary diagnostic criterion for sarcopenia (Cruz-Jentoft et al., 2019); however, muscle strength is superior to muscle mass in predicting adverse outcomes of sarcopenia. Additionally, some studies suggest that increased TNF-α secretion during the early stages of muscle injury can promote muscle repair (Dumont et al., 2015). The dual role of TNF-α may also depend on temporal and quantitative factors. Transient elevation postinjury supports muscle repair via satellite cell activation. However, sustained muscle damage leads to a continuous increase in TNF-α secretion by immune cells, which further damages the muscle (Wang, 2022).

Although some studies have presented conflicting conclusions, it remains undeniable that muscle mass, muscle strength, and physical function are associated with inflammation in older adults to varying degrees. However, whether elevated levels of TNF-α can serve as a diagnostic criterion for sarcopenia requires clarification in future in-depth research.

### 4.3 CRP

CRP, a pentameric acute-phase protein, serves as a well-established inflammatory biomarker (Del Giudice and Gangestad, 2018). CRP, which is primarily synthesized by the liver in response to trauma, chronic disease, or IL-6 stimulation, is also produced by smooth muscle cells, macrophages, endothelial cells, lymphocytes, and adipocytes (Sproston and Ashworth, 2018).

High-sensitivity CRP (hs-CRP) enables the detection of low-grade inflammation with enhanced sensitivity during subclinical stages. Elevated baseline CRP is consistently associated with cardiovascular events, stroke, malignancies, age-related degeneration, and mortality (Pope and Choy, 2021; Rizo-Téllez et al., 2023). Multiple studies have linked both CRP and hs-CRP to sarcopenia. Mechanistically, CRP may induce insulin resistance and modulate protein kinase B (Akt) signaling, leading to myocyte dysfunction, reduced fiber size, and diminished muscle strength (Wåhlin-Larsson et al., 2017).

In mouse models, moderate CRP elevation impaired skeletal muscle glucose uptake via Fc gamma receptors IIB (FcγRIIB)-mediated endothelial insulin resistance (Tanigaki et al., 2016), subsequently suppressing MPS and promoting proteolysis (Lu et al., 2022). Clinically, a cross-sectional study of 4,252 men aged 60–79 years demonstrated that elevated CRP (median 1.8 mg/L, IQR 0.8–3.8, in the lowest muscle mass quartile) was correlated with reduced muscle mass, independent of age and body composition (Atkins et al., 2014). An aging cohort study revealed that increased CRP (median 2.3 mg/L in the overall group) and IL-6 (median 2.3 pg/mL in the overall group) levels were associated with poorer cognitive/functional performance and reduced survival (Puzianowska-Kuźnicka et al., 2016). Another cross-sectional outpatient study confirmed significantly higher CRP levels in sarcopenic elderly individuals (median 10.1 mg/dL vs. 3.9 mg/dL in non-sarcopenic controls), along with correlations between erythrocyte sedimentation rate (ESR; mean 41.3 mm/s vs. 18.8 mm/s), adiponectin (median 6.0 μg/mL vs. 9.6 μg/mL), and sarcopenia (Can et al., 2017). A meta-analysis of 19 cross-sectional studies further revealed inverse correlations between CRP/hs-CRP and muscle strength (Shokri-Mashhadi et al., 2021). However, conflicting evidence exists. A multicenter prospective cohort reported no significant associations between hs-CRP (median 0.231 mg/L, IQR 0.118–0.475 mg/L) and sarcopenia parameters despite correlations with reduced physical activity (Dupont et al., 2021). An umbrella review noted inconsistent statistical significance for CRP elevation in sarcopenia across studies, attributing heterogeneity to population characteristics and diagnostic criteria. The current evidence quality for CRP as a sarcopenia biomarker remains “very low,” precluding its diagnostic use alone (Liu G. et al., 2025).

In summary, while CRP is frequently elevated in sarcopenic patients, existing evidence cannot establish causality. CRP alone is inadequate for the diagnosis of sarcopenia, and its combined use with other inflammatory markers requires further rigorous validation.

### 4.4 IL-10

IL-10 is a well-characterized anti-inflammatory cytokine produced by macrophages, helper T cells, B cells, and monocytes. It modulates inflammatory and immune responses primarily by suppressing monocyte and macrophage functions while inhibiting T-cell production of proinflammatory cytokines such as IFN-γ, TNF, IL-1, and IL-6 (Nagata and Nishiyama, 2021). Through these mechanisms, IL-10 alleviates chronic low-grade inflammation (Ouyang and O’Garra, 2019), highlighting its research relevance in sarcopenia.

In an IL-10 systemic knockout mouse model, the absence of IL-10 significantly impaired the polarization of macrophages toward the proregenerative M2 phenotype (CD206^+^/CD163^+^) following acute muscle injury without affecting overall macrophage recruitment. This establishes IL-10 as an essential regulator of muscle repair (Welc et al., 2020). Similarly, in a rat model of volumetric muscle loss, sustained local IL-10 delivery improved functional and structural recovery by modulating immune cell activity, particularly that of Tregs, promoting angiogenesis, and extending the myogenic regeneration window, supporting its potential as an immunotherapeutic adjunct in regenerative strategies (Huynh et al., 2023). A cross-sectional study of 164 adults aged 61–90 years reported elevated levels of IL-6 (43.80 ± 10.13 pg/mL vs. 27.38 ± 9.53 pg/mL in non-sarcopenic controls) and IL-10 (4.13 ± 1.03 pg/mL vs. 3.75 ± 1.21 pg/mL) and an increased IL-6/IL-10 ratio (9.71 ± 1.43 vs. 9.09 ± 1.71) in elderly subjects with sarcopenia. IL-10 correlated positively with age, suggesting that its increase may represent a compensatory anti-inflammatory response aimed at countering elevated IL-6. However, the compensatory increase in IL-10 appears insufficient to fully neutralize IL-6-mediated inflammation, maintaining a proinflammatory state (Rong et al., 2018). As a cross-sectional analysis, this study cannot establish causality, warranting validation through prospective trials.

In summary, IL-10 has the capacity to suppress key proinflammatory pathways involved in sarcopenia, positioning it as a potential target for novel intervention strategies.

### 4.5 IL-15

IL-15, a T-cell growth factor produced mainly by monocytes and macrophages, shares functional similarities with IL-2. Its mRNA is expressed in multiple tissues, particularly skeletal muscle and placenta, where it contributes to immune cell growth and differentiation. Growing evidence supports its role as a myokine that modulates skeletal muscle metabolism, mitochondrial biogenesis, and energy supply (Tagliaferri et al., 2015), ultimately contributing to muscle function.

Multiple studies have highlighted the beneficial effects of IL-15 on skeletal muscle (O’Leary et al., 2017). Krolopp et al. reported that IL-15 activates the JAK3/STAT3 pathway, promoting glucose transporter type 4 (GLUT4) translocation to the cell membrane and enhancing glucose uptake in muscle cells, suggesting a metabolic role relevant to diabetes (Krolopp et al., 2016). Kang et al. reported that IL-15 stimulates fibroblast-associated protein (FAP) proliferation via JAK/STAT signaling, inhibits adipogenic differentiation, and improves muscle regeneration and fiber quality (Kang et al., 2018). Using primary human skeletal muscle myoblasts as an *in vitro* model, O'Leary et al. demonstrated that IL-15 significantly increased myotube thickness and nuclear fusion, promoting muscle formation. A previous study revealed that TNF-α stimulation triggers skeletal muscle cells to upregulate both the expression and secretion of IL-15 and its receptors (O’Leary et al., 2017). These findings indicate that IL-15 can counteract inflammation-related muscle loss, suggesting its potential as a therapeutic target for reducing inflammation-mediated skeletal muscle atrophy. However, some studies question its anabolic role. IL-15 administration in healthy rats did not increase muscle mass, and transgenic mice overexpressing IL-15 presented no hypertrophy (Pistilli and Quinn, 2013), suggesting that IL-15 may function primarily as a regulator of oxidative stress and fatigue resistance rather than as a direct anabolic agent. Aging models show reduced muscle IL-15 expression alongside elevated TNF-α and serum phosphorus (Alcalde-Estévez et al., 2025). Clinically, the level of circulating IL-15 decreases with age (Duggal et al., 2018), suggesting that its loss may contribute to sarcopenia (LeDrew et al., 2025). A cross-sectional study of 160 older adults linked low plasma IL-15 (median 3.91 pg/mL vs. 5.1 pg/mL in controls) to sarcopenia (Yalcin et al., 2018), although its dynamic changes and utility as a biomarker or therapeutic target require further prospective investigation.

In summary, IL-15 has favorable effects on muscle metabolism and inflammation, supporting its potential as a therapeutic target for sarcopenia. However, its exact role across physiological and pathological contexts remains to be fully elucidated.

## 5 Targeted interventions for sarcopenia and associated inflammation

### 5.1 Exercise intervention

Exercise training represents one of the most effective nonpharmacological interventions for modifying functional outcomes in older adults. Evidence indicates that physical activity beneficially influences multiple systems involved in the aging process, including skeletal muscle (Angulo et al., 2020). A time-substitution model in a community-dwelling older adult cohort demonstrated that moderate-to-vigorous physical activity is associated with greater muscle mass, faster walking speed, and greater grip strength, along with a significantly lower prevalence of sarcopenia. However, these benefits appear to be subject to an intensity threshold beyond which additional gains diminish. Moreover, exercise exceeding 1.5 h reaches a point of oversaturation, yielding only marginal improvements in physical performance (Sánchez-Sánchez et al., 2019). A meta-analysis further confirmed that physical exercise significantly enhances muscle strength, gait speed, and the skeletal muscle mass index in sarcopenic individuals, supporting its role as a core therapeutic strategy (Zhang et al., 2021).

Resistance training (RE), in particular, is widely regarded as the optimal exercise modality for improving muscle mass and strength. Current clinical guidelines strongly recommend RE as a first-line intervention for sarcopenia (Dent et al., 2018; Fragala et al., 2019). A well-structured progressive resistance training program induces positive neuromuscular adaptations and markedly increases muscle mass and strength (Hurst et al., 2022). A 12-week trial comparing light-versus heavy-load RE in older adults demonstrated that both regimens similarly improved maximal strength and promoted muscle hypertrophy (Rodriguez-Lopez et al., 2022).

In addition to its direct benefits, RE may also mitigate sarcopenia-related inflammation. Moderate exercise mitigates obesity-related chronic inflammation by suppressing key inflammatory pathways such as NF-κB and the NLRP3 inflammasome, thereby reducing the expression and release of pro-inflammatory cytokines, including TNF-α, IL-6, and IL-1β in skeletal muscle (Liu and Chang, 2018; Gomarasca et al., 2022). Resistance and combined exercise regimens further modulate systemic inflammation through the regulation of myokine and adipokine secretion. Specifically, these interventions elevate levels of anabolic mediators such as IGF-1 and irisin, promoting muscle protein synthesis (Shi et al., 2017; Paris et al., 2020), while also increasing adiponectin and ameliorating leptin resistance, which collectively improve inflammatory profiles (Gaspar et al., 2018; Li et al., 2019). Additionally, exercise enhances mitochondrial quality control, attenuating oxidative stress and inflammatory signaling, and influences the expression of specific microRNAs that repress transcription of inflammation-related genes (Russell et al., 2017; Marcangeli et al., 2022). Together, these interconnected mechanisms underlie the anti-inflammatory effects of exercise, which have been consistently associated with reduced circulating inflammatory markers in clinical studies. A *post hoc*analysis of a randomized controlled trial involving 57 patients with sarcopenia and 57 matched controls revealed that after 12 weeks of resistance training combined with nutritional support intervention, patients demonstrated significant improvements in muscle mass and grip strength, along with marked reductions in serum TNF-α, IL-1β, and IL-6 levels. This indicates that combined exercise and nutritional intervention can simultaneously enhance muscle function and reduce systemic inflammation in patients with sarcopenia (Chang et al., 2023). Regular exercise has been shown to reduce the levels of proinflammatory cytokines and CRP (Paolucci et al., 2018; Sadjapong et al., 2020). A meta-analysis of randomized controlled trials confirmed that RE significantly lowers CRP and tends to reduce IL-6 in older adults, supporting its role in countering low-grade inflammation (Sardeli et al., 2018). However, no significant effect on TNF-α was observed, possibly due to the limited number of studies and population heterogeneity. A separate systematic review indicated that exercise at any intensity can reduce chronic inflammation, although high-intensity training may be more effective in middle-aged adults (Rose et al., 2021). Animal studies have revealed that RE attenuates muscle atrophy by modulating the Akt/FoxO1 signaling pathway and that its benefits are enhanced when RE is combined with anti-inflammatory nutrition (Sumi et al., 2020).

In addition to RE, aerobic exercise also confers benefits in the management of sarcopenia. A meta-analysis reported that aerobic exercise lowers systemic levels of IL-6, TNF-α, and CRP in older adults (Zheng et al., 2019). It improves exercise tolerance and facilitates adaptation to resistance training, creating a virtuous cycle of physical improvement. Combined resistance, aerobic, and balance training is considered one of the most effective integrated approaches for treating sarcopenia and enhancing quality of life in elderly individuals (Shen et al., 2023).

### 5.2 Nutritional intervention

Malnutrition and the subsequent decline in MPS are significant contributors to and predictors of sarcopenia onset and progression (Beaudart et al., 2019) but also serve as key targets for intervention. Nutrients and dietary patterns have demonstrated protective effects on muscle tissue, helping to counteract age-related loss of strength and function (Ligthart-Melis et al., 2020).

Adequate protein intake enhances amino acid utilization by activating mammalian target of rapamycin (mTOR) signaling and its downstream targets, thereby stimulating the synthesis of myoplasmic and myofibrillar proteins (Hollingworth et al., 2021). Increasing dietary protein intake effectively mitigates age-related muscle decline and slows the progression of sarcopenia (Li et al., 2021). A randomized controlled trial involving 50 sarcopenic patients aged ≥65 years reported that leucine supplementation significantly improved walking performance and body mass index, although no significant changes in IL-6 or TNF-α levels were observed18 (Martínez-Arnau et al., 2020). Other studies support that leucine-rich or whey protein supplementation effectively increases muscle mass and moderately improves muscle function (Cereda et al., 2022; Zhou et al., 2024). In a 12-week trial with 120 mildly frail older adults, higher-dose whey protein supplementation significantly increased appendicular muscle mass compared with lower doses (Park et al., 2018). Furthermore, one randomized controlled trial found that combining resistance training with whey protein results in more substantial improvements in muscle mass than exercise alone (Yamada et al., 2019). β-Hydroxy-β-methylbutyrate (HMB), a leucine metabolite with anabolic properties, has been shown to be effective in enhancing muscle health in older adults at risk of sarcopenia, particularly in improving limb muscle strength (Bear et al., 2019). In addition to its direct effects on skeletal muscle, animal studies have demonstrated that HMB supplementation can reduce levels of proinflammatory markers such as IL-6, IL-1β, TNF-α, and CXCL2 in colonic tissue by inhibiting ERK/NF-κB activation in macrophages, thereby improving DSS-induced colitis (Liu J. et al., 2025).

Vitamin D influences skeletal muscle both indirectly through calcium‒phosphorus homeostasis and directly via effects on muscle cell proliferation and differentiation, promoting type II muscle fiber hypertrophy (Bass et al., 2020). A randomized trial revealed that cholecalciferol supplementation for 6 months improved muscle mass in sarcopenic patients with vitamin D deficiency (El Hajj et al., 2018). Mechanistically, vitamin D appears to reduce the expression of the atrophy-related proteins MuRF1, muscle atrophy F-box (MAFbx), and forkhead box O3 (FOXO3a) (Yang et al., 2020). An animal model study found that in male subjects, vitamin D effectively suppressed NF-κB pathway activation, reduced expression of pro-inflammatory factors such as IL-1β, IL-6, and TNF-α, and decreased genes associated with muscle atrophy. This indicates that vitamin D supplementation can improve sarcopenia by lowering skeletal muscle inflammation levels (Yang et al., 2025). However, evidence regarding the efficacy of vitamin D in improving muscle strength remains inconsistent, potentially due to variations in supplementation protocols and the complexity of its actions in muscle tissue (Abshirini et al., 2020). Furthermore, the optimal dosage, dosing frequency, or treatment duration for improving muscle mass or function remains unclear, necessitating further research in this area (Uchitomi et al., 2020).

n-3 and n-6 polyunsaturated fatty acids, along with conjugated linoleic acid (CLA), not only delay the onset of sarcopenia by directly influencing muscle protein synthesis and degradation, but also intervene in sarcopenia by reducing inflammatory responses. Randomized trials have demonstrated that eicosapentaenoic acid (EPA) and docosahexaenoic acid (DHA) supplementation reduces IL-6, IL-1β, and TNF-α levels in older adults. Omega-3 polyunsaturated fatty acids (PUFAs) may also exert anabolic effects by activating mTOR signaling and improving insulin sensitivity (Smith et al., 2015; Dupont et al., 2019). Other studies also suggest that omega-3 supplementation can effectively improve protein synthesis, muscle mass, and muscle function (Gray and Mittendorfer, 2018). Additional evidence indicates that they enhance mitochondrial function by reducing ROS production, thereby potentially delaying age-related muscle loss (Troesch et al., 2020). A cross-sectional study found that n-6 polyunsaturated fatty acids showed a strong negative correlation with the key inflammatory marker CRP (Virtanen et al., 2018). Animal models have also demonstrated that CLA supplementation can prevent bone and muscle mass loss by regulating markers of inflammation and osteoclast factors (Rahman et al., 2007).

In addition to specific nutrients, overall dietary patterns significantly influence sarcopenia risk. The dietary inflammatory index (DII) can be used to quantify the inflammatory potential of diets (Shivappa et al., 2014). Proinflammatory diets (high DII scores), rich in refined carbohydrates and saturated fats, promote systemic inflammation (Minihane et al., 2015), whereas anti-inflammatory diets (low DII scores), containing PUFAs, dietary fiber, and flavonoids, reduce inflammation (Poulsen et al., 2020). A meta-analysis revealed that each one-point increase in DII score was associated with a 1.22-fold increase in sarcopenia incidence (Diao et al., 2023), suggesting that shifting toward anti-inflammatory dietary patterns may help prevent or delay sarcopenia onset.

### 5.3 Combined exercise and nutrition intervention

Compared with single-modality approaches, combined exercise and nutrition interventions produce more consistent and superior outcomes. In multiple randomized controlled trials (RCTs) involving community-dwelling Japanese women with sarcopenia, KIM et al. demonstrated that supplementation with amino acids and tea catechins, both independently and combined with exercise, significantly improved body composition and physical function (Kim et al., 2016). A 24-week RCT revealed that whey protein supplementation following resistance exercise increased muscle mass, grip strength, and walking speed in older women, effectively preventing sarcopenia (Mori and Tokuda, 2018). Another RCT in sarcopenic elderly women confirmed that combining whey protein with resistance training increased appendicular lean soft tissue, reduced total and trunk fat mass, and decreased the risk of sarcopenia and sarcopenic obesity, although the anti-inflammatory effects remain limited (Nabuco et al., 2019).

The 2019 Asian Working Group for Sarcopenia (AWGS) Consensus noted that combined nutrition and exercise therapies consistently improve muscle strength and physical function, although the effects on muscle mass vary (Chen et al., 2020). Both the EWGSOP and the International Conference on Frailty and Sarcopenia Research (ICFSR) acknowledge the superiority of integrated interventions over isolated nutritional or exercise approaches. While substantial evidence supports the efficacy of combined interventions, further high-quality research is needed owing to the small sample sizes and potential biases in existing studies.

## 6 Pharmacological interventions for sarcopenia

### 6.1 Traditional medicine

No medications have yet been approved by the U.S. Food and Drug Administration specifically for sarcopenia treatment. Current pharmacological approaches aim to improve muscle mass and strength through agents such as testosterone, selective androgen receptor modulators (SARMs), estrogen, DHEA, IGF-1, growth hormone (GH), growth hormone-releasing hormone (GHRH), myostatin, activin receptor pathway inhibitors, angiotensin-converting enzyme inhibitors (ACEis), angiotensin receptor blockers (ARBs), and beta-blockers (Rolland et al., 2023), although their therapeutic efficacy varies considerably.

According to the Belgian Society of Geriatrics and Gerontology (BSGG), testosterone supplementation significantly increases muscle mass in men with low serum testosterone levels, although its effect on muscle strength remains limited (Gielen et al., 2021). Several reviews and meta-analyses have indicated that testosterone improves body composition in older hypogonadal men by increasing lean mass and reducing fat mass (Snyder et al., 2016; Storer et al., 2017; Midttun et al., 2024). However, its impact on physical performance and exercise capacity remains uncertain. Substantial heterogeneity in study designs, including population characteristics, administration routes, dosages, and outcome measures, limits comparability across trials, warranting further validation of its clinical utility. Additionally, studies indicate that testosterone therapy fails to reduce the incidence of clinical fractures in elderly men and may even increase the risk of atypical fractures (Snyder et al., 2024). Concurrently, the treatment carries well-documented safety concerns, including polycythemia, exacerbation of benign prostatic hyperplasia, and potential risks associated with prostate cancer (Qaseem et al., 2020). Clinical decisions must be strictly limited to patients with symptomatic hypogonadism, and treatment should only be administered after thorough risk disclosure and under close monitoring.

SARMs have demonstrated anabolic effects on bone and muscle tissue without stimulating nonskeletal tissues. Preclinical and phase II clinical studies in older adults and cancer patients indicate that SARMs improve muscle mass and function (Ponnusamy et al., 2017; Mohan et al., 2023). SARMs do not bind to progesterone or glucocorticoid receptors, suggesting potential therapeutic prospects for women with sarcopenia (Rolland et al., 2023). Unlike testosterone, it has shown efficacy in enhancing muscle mass, strength, and physical function (Mohan et al., 2023), with encouraging results reported in patients with muscle wasting associated with severe inflammatory conditions. Notwithstanding their potential to enhance muscle mass and strength in preliminary investigations, the therapeutic application of SARMs for sarcopenia faces substantial criticism (Rolland et al., 2023). Crucially, robust evidence linking these biochemical improvements to clinically meaningful outcomes, such as enhanced physical function or quality of life in the elderly remains absent. Significant safety concerns, particularly a documented risk of drug-induced liver injury, further complicate their profile for a condition necessitating long-term management (Vignali et al., 2023). Consequently, the long-term risk-benefit ratio of SARMs is presently indeterminate, and their establishment as a standard clinical intervention for sarcopenia appears distant.

Evidence regarding the efficacy of pharmacological agents, including estrogen, DHEA, IGF-1, GH, GHS, ACEi, ARB, and beta-blockers, in sarcopenia remains limited or absent. However, their effects on muscle mass, strength, and physical performance are inconsistent, highlighting the need for targeted clinical trials to evaluate their potential role in sarcopenia management.

### 6.2 Anti-inflammatory drugs

Given the established link between sarcopenia and chronic inflammation, age-related inflammatory processes significantly contribute to its development and progression. Consequently, therapeutic strategies targeting chronic inflammation have become a research hotspot and major trend.

Systemic anti-inflammatory medications, particularly nonsteroidal anti-inflammatory drugs (NSAIDs), may help preserve muscle mass and function. Compared with nonusers, regular NSAID users demonstrate an approximately 80% lower risk of developing sarcopenia, suggesting potential benefits in mitigating inflammation-related muscle deterioration (Landi et al., 2013). Theophylline, a methylxanthine derivative used in chronic obstructive pulmonary disease (COPD) management, has been shown to reduce the circulating levels of IL-6 and TNF (Allen, 2017). While these findings support its potential anti-inflammatory properties, no studies have confirmed its efficacy specifically for the treatment of sarcopenia.

In addition to systemic anti-inflammatories, targeted therapies against specific cytokines and inflammatory pathways represent promising approaches. In mouse models, the TNF-α inhibitor etanercept prevents type II fiber atrophy and improves muscle function (Sciorati et al., 2020). Similarly, infliximab (IFX), an anti-TNF antibody, increases muscle strength and volume in Crohn’s disease patients, reversing inflammatory muscle wasting (Subramaniam et al., 2015). Additionally, current research has developed machine learning models based on CT radiomics, utilizing psoas major muscle volume as a surrogate marker for skeletal muscle mass to predict the response to IFX therapy effectively in Crohn’s disease patients (Chen et al., 2025). Tocilizumab (TCZ), an IL-6 receptor antagonist, increased lean body mass in rheumatoid arthritis patients after 1 year of treatment, primarily through the redistribution of adipose tissue to subcutaneous compartments (Tournadre et al., 2017).

Currently, there are few clinical studies on the efficacy of inflammatory factor inhibitors in treating sarcopenia patients. The NF-κB pathway, a key regulator of inflammation and muscle atrophy, represents another therapeutic target. Its inhibition suppresses TNF-α-induced upregulation of MuRF1, thereby preventing muscle wasting (Thoma and Lightfoot, 2018). In mouse models, reducing tumor necrosis factor receptor-associated factor 6 (TRAF6) expression rescued myofibrillar degradation and preserved muscle fiber size and strength (Mourkioti et al., 2006). JAK/STAT pathway inhibition has been shown to reduce SASP in aged adipose tissue, decrease systemic inflammation, and improve muscle function (Xu et al., 2015; Thoma and Lightfoot, 2018). Additionally, mTOR pathway inhibition appears to be beneficial by reducing muscle fibrosis, enhancing regeneration, and extending healthspan, despite chronic mTOR activation being associated with muscle atrophy (Liu and Sabatini, 2020).

In summary, given the link between sarcopenia and chronic inflammation, targeting the inhibition of inflammatory cytokines and pathways represents a novel therapeutic direction for sarcopenia. However, preclinical studies and evidence from patients with chronic inflammatory diseases with secondary sarcopenia suggest that inhibiting specific cytokines or their downstream signaling pathways can effectively improve muscle mass and function, translating this approach to the larger population of elderly individuals with primary sarcopenia faces fundamental limitations. To date, nearly all positive clinical evidence comes from patients with secondary sarcopenia and clear, active systemic inflammation. High-quality randomized controlled trials are lacking to determine whether these benefits extend to the general sarcopenic elderly population, which is characterized by relatively low-grade inflammation and high heterogeneity.Systemic anti-inflammatory therapies, particularly biologics such as TNF-α inhibitors and JAK inhibitors, entail substantial immunosuppressive risks, including severe infections, activation of opportunistic pathogens, and potential oncogenic effects. For older adults with age-related immunosenescence, the long-term risk–benefit profile remains highly uncertain. Moreover, achieving tissue-selective or moderate inhibition of key targets such as NF-κB and mTOR which play pleiotropic roles in systemic physiology without inducing systemic toxicity remains a major technical hurdle.

Thus, despite promising mechanistic insights, systemic anti-inflammatory drugs cannot yet be considered safe or established treatments for primary sarcopenia. Future research should prioritize the identification of patient subgroups, defined by specific biomarkers, that are most likely to benefit from anti-inflammatory interventions. Concurrently, safer and more targeted strategies warrant further exploration.

## 7 Conclusion

Sarcopenia is an age-related geriatric syndrome characterized by the progressive loss of skeletal muscle mass and quality throughout the body, with or without functional impairment. It may lead to adverse outcomes such as functional decline, frailty, falls, hospitalization, disability, and even death, posing a serious threat to the health and wellbeing of elderly individuals. Increasing research indicates that inflammatory aging, a state of persistent, low-grade chronic inflammation that progresses with age in the absence of infection, is a key mechanism underlying sarcopenia. Chronic inflammation driven by cellular senescence, immune aging, oxidative stress and mitochondrial dysfunction, altered hormone levels, gut microbiota dysbiosis, and obesity contributes to the development and progression of sarcopenia. In obese elderly individuals, aging adipose tissue forms a vicious cycle with muscle tissue through shared inflammatory pathways. This process not only contributes to sarcopenia but also leads to a mixed condition characterized by reduced muscle mass and quality alongside obesity. Scholars have proposed a new definition for this phenomenon: sarcopenic obesity. At the molecular level of inflammation, alterations in inflammatory cytokines and their signaling pathways influence skeletal muscle synthesis, degradation, and myofibrillar remodeling. However, research on the impact of these molecules on sarcopenia remains nascent, and prospective, large-scale cohort studies or RCTs are needed to validate these serum biomarkers as independent, sensitive markers of sarcopenia. Current management strategies for sarcopenia encompass exercise, nutritional support, combined exercise-nutritional approaches, and pharmacological interventions. At present, resistance training and nutritional optimization constitute the cornerstone of management, exerting direct benefits on muscle mass and function while concurrently modulating underlying chronic low-grade inflammation. Given the pathophysiological link between inflammation and sarcopenia, targeting inflammatory pathways through pharmacological agents has emerged as a novel therapeutic avenue, highlighting a promising future direction for interventions aimed at mitigating the onset and progression of the condition. We anticipate that future therapies will effectively improve the occurrence and progression of sarcopenia.

## References

[B1] Abshirini M. Mozaffari H. Kord-Varkaneh H. Omidian M. Kruger M. C. (2020). The effects of vitamin D supplementation on muscle strength and mobility in postmenopausal women: a systematic review and meta-analysis of randomised controlled trials. J. Hum. Nutr. Diet. 33, 207–221. 10.1111/jhn.12717 31729817

[B2] Adachi H. Fujimoto K. Fujii A. Yamasaki K. Okada K. Matsuura T. (2020). Long-term retrospective observation study to evaluate effects of adiponectin on skeletal muscle in renal transplant recipients. Sci. Rep. 10, 10723. 10.1038/s41598-020-67711-1 32612097 PMC7330033

[B3] Alcalde-Estévez E. Moreno-Piedra A. Asenjo-Bueno A. Martos-Elvira M. de la Serna-Soto M. Ruiz-Ortega M. (2025). Aging-related hyperphosphatemia triggers the release of TNF-α from macrophages, promoting indicators of sarcopenia through the reduction of IL-15 expression in skeletal muscle. Life Sci. 368, 123507. 10.1016/j.lfs.2025.123507 40010633

[B4] Allen S. C. (2017). Systemic inflammation in the genesis of frailty and sarcopenia: an overview of the preventative and therapeutic role of exercise and the potential for drug treatments. Geriatr. (Basel) 2, 6. 10.3390/geriatrics2010006 31011016 PMC6371169

[B5] Angulo J. El Assar M. Rodríguez-Mañas L. (2016). Frailty and sarcopenia as the basis for the phenotypic manifestation of chronic diseases in older adults. Mol. Asp. Med. 50, 1–32. 10.1016/j.mam.2016.06.001 27370407

[B6] Angulo J. El Assar M. Álvarez-Bustos A. Rodríguez-Mañas L. (2020). Physical activity and exercise: strategies to manage frailty. Redox Biol. 35, 101513. 10.1016/j.redox.2020.101513 32234291 PMC7284931

[B7] Atkins J. L. Whincup P. H. Morris R. W. Wannamethee S. G. (2014). Low muscle mass in older men: the role of lifestyle, diet and cardiovascular risk factors. J. Nutr. Health Aging 18, 26–33. 10.1007/s12603-013-0336-9 24402385 PMC12878516

[B8] Baechle J. J. Chen N. Makhijani P. Winer S. Furman D. Winer D. A. (2023). Chronic inflammation and the hallmarks of aging. Mol. Metab. 74, 101755. 10.1016/j.molmet.2023.101755 37329949 PMC10359950

[B9] Bano G. Trevisan C. Carraro S. Solmi M. Luchini C. Stubbs B. (2017). Inflammation and sarcopenia: a systematic review and meta-analysis. Maturitas 96, 10–15. 10.1016/j.maturitas.2016.11.006 28041587

[B10] Bartke A. (2021). Growth hormone and aging. Rev. Endocr. Metab. Disord. 22, 71–80. 10.1007/s11154-020-09593-2 33001358

[B11] Bass J. J. Nakhuda A. Deane C. S. Brook M. S. Wilkinson D. J. Phillips B. E. (2020). Overexpression of the vitamin D receptor (VDR) induces skeletal muscle hypertrophy. Mol. Metab. 42, 101059. 10.1016/j.molmet.2020.101059 32771696 PMC7475200

[B12] Batsis J. A. Villareal D. T. (2018). Sarcopenic obesity in older adults: aetiology, epidemiology and treatment strategies. Nat. Rev. Endocrinol. 14, 513–537. 10.1038/s41574-018-0062-9 30065268 PMC6241236

[B13] Baumgartner R. N. (2000). Body composition in healthy aging. Ann. N. Y. Acad. Sci. 904, 437–448. 10.1111/j.1749-6632.2000.tb06498.x 10865787

[B14] Bear D. E. Langan A. Dimidi E. Wandrag L. Harridge S. D. R. Hart N. (2019). β-Hydroxy-β-methylbutyrate and its impact on skeletal muscle mass and physical function in clinical practice: a systematic review and meta-analysis. Am. J. Clin. Nutr. 109, 1119–1132. 10.1093/ajcn/nqy373 30982854

[B15] Beaudart C. Sanchez-Rodriguez D. Locquet M. Reginster J.-Y. Lengelé L. Bruyère O. (2019). Malnutrition as a strong predictor of the onset of sarcopenia. Nutrients 11, 2883. 10.3390/nu11122883 31783482 PMC6950107

[B16] Belizário J. E. Fontes-Oliveira C. C. Borges J. P. Kashiabara J. A. Vannier E. (2016). Skeletal muscle wasting and renewal: a pivotal role of myokine IL-6. Springerplus 5, 619. 10.1186/s40064-016-2197-2 27330885 PMC4870483

[B17] Bennett J. L. Pratt A. G. Dodds R. Sayer A. A. Isaacs J. D. (2023). Rheumatoid sarcopenia: loss of skeletal muscle strength and mass in rheumatoid arthritis. Nat. Rev. Rheumatol. 19, 239–251. 10.1038/s41584-023-00921-9 36801919

[B18] Bhanji R. A. Montano-Loza A. J. Watt K. D. (2019). Sarcopenia in cirrhosis: looking beyond the skeletal muscle loss to see the systemic disease. Hepatology 70, 2193–2203. 10.1002/hep.30686 31034656

[B19] Bian A. Ma Y. Zhou X. Guo Y. Wang W. Zhang Y. (2020). Association between sarcopenia and levels of growth hormone and insulin-like growth factor-1 in the elderly. BMC Musculoskelet. Disord. 21, 214. 10.1186/s12891-020-03236-y 32264885 PMC7140321

[B20] Bilski J. Pierzchalski P. Szczepanik M. Bonior J. Zoladz J. A. (2022). Multifactorial mechanism of sarcopenia and sarcopenic obesity. Role of physical exercise, microbiota and myokines. Cells 11, 160. 10.3390/cells11010160 35011721 PMC8750433

[B21] Birch J. Gil J. (2020). Senescence and the SASP: many therapeutic avenues. Genes Dev. 34, 1565–1576. 10.1101/gad.343129.120 33262144 PMC7706700

[B22] Bossi P. Delrio P. Mascheroni A. Zanetti M. (2021). The spectrum of malnutrition/cachexia/sarcopenia in oncology according to different cancer types and settings: a narrative review. Nutrients 13, 1980. 10.3390/nu13061980 34207529 PMC8226689

[B23] Brorson J. Lin L. Wang J. Bæk A. Billeskov T. B. Thybo F. F. (2025). Complementing muscle regeneration-fibro-adipogenic progenitor and macrophage-mediated repair of elderly human skeletal muscle. Nat. Commun. 16, 5233. 10.1038/s41467-025-60627-2 40473693 PMC12141666

[B24] Bunnell B. A. (2021). Adipose tissue-derived mesenchymal stem cells. Cells 10, 3433. 10.3390/cells10123433 34943941 PMC8700397

[B25] Calcinotto A. Kohli J. Zagato E. Pellegrini L. Demaria M. Alimonti A. (2019). Cellular senescence: aging, cancer, and injury. Physiol. Rev. 99, 1047–1078. 10.1152/physrev.00020.2018 30648461

[B26] Can B. Kara O. Kizilarslanoglu M. C. Arik G. Aycicek G. S. Sumer F. (2017). Serum markers of inflammation and oxidative stress in sarcopenia. Aging Clin. Exp. Res. 29, 745–752. 10.1007/s40520-016-0626-2 27571781

[B27] Cao L. Morley J. E. (2016). Sarcopenia is recognized as an independent condition by an international classification of disease, tenth revision, clinical modification (ICD-10-CM) code. J. Am. Med. Dir. Assoc. 17, 675–677. 10.1016/j.jamda.2016.06.001 27470918

[B28] Cereda E. Pisati R. Rondanelli M. Caccialanza R. (2022). Whey protein, Leucine- and Vitamin-D-Enriched oral nutritional supplementation for the treatment of sarcopenia. Nutrients 14, 1524. 10.3390/nu14071524 35406137 PMC9003251

[B29] Chang K.-V. Wu W.-T. Chen Y.-H. Chen L.-R. Hsu W.-H. Lin Y.-L. (2023). Enhanced serum levels of tumor necrosis factor-α, interleukin-1β, and -6 in sarcopenia: alleviation through exercise and nutrition intervention. Aging (Albany NY) 15, 13471–13485. 10.18632/aging.205254 38032288 PMC10713395

[B30] Chen L.-K. Woo J. Assantachai P. Auyeung T.-W. Chou M.-Y. Iijima K. (2020). Asian working group for sarcopenia: 2019 consensus update on sarcopenia diagnosis and treatment. J. Am. Med. Dir. Assoc. 21, 300–307.e2. 10.1016/j.jamda.2019.12.012 32033882

[B31] Chen X. Feng J. Chang Q. Lu F. Yuan Y. (2021a). Senescence of donor cells impairs fat graft regeneration by suppressing adipogenesis and increasing expression of senescence-associated secretory phenotype factors. Stem Cell. Res. Ther. 12, 311. 10.1186/s13287-021-02383-w 34051860 PMC8164816

[B32] Chen Z. Li W.-Y. Ho M. Chau P.-H. (2021b). The prevalence of sarcopenia in Chinese older adults: meta-analysis and meta-regression. Nutrients 13, 1441. 10.3390/nu13051441 33923252 PMC8146971

[B33] Chen Z. Cai W. He Y. Mei T. Zhang Y. Li S. (2025). Psoas muscle CT radiomics-based machine learning models to predict response to infliximab in patients with Crohn’s disease. Ann. Med. 57, 2527954. 10.1080/07853890.2025.2527954 40616584 PMC12231329

[B34] Cheng X. Jiang S. Pan B. Xie W. Meng J. (2023). Ectopic and visceral fat deposition in aging, obesity, and idiopathic pulmonary fibrosis: an interconnected role. Lipids Health Dis. 22, 201. 10.1186/s12944-023-01964-3 38001499 PMC10668383

[B35] Choi H. M. Doss H. M. Kim K. S. (2020). Multifaceted physiological roles of adiponectin in inflammation and diseases. Int. J. Mol. Sci. 21, 1219. 10.3390/ijms21041219 32059381 PMC7072842

[B36] Clegg A. Hassan-Smith Z. (2018). Frailty and the endocrine system. Lancet Diabetes Endocrinol. 6, 743–752. 10.1016/S2213-8587(18)30110-4 30017798

[B37] Clemente J. C. Manasson J. Scher J. U. (2018). The role of the gut microbiome in systemic inflammatory disease. BMJ 360, j5145. 10.1136/bmj.j5145 29311119 PMC6889978

[B38] Cruz-Jentoft A. J. Bahat G. Bauer J. Boirie Y. Bruyère O. Cederholm T. (2019). Sarcopenia: revised European consensus on definition and diagnosis. Age Ageing 48, 16–31. 10.1093/ageing/afy169 30312372 PMC6322506

[B39] de Sire R. Rizzatti G. Ingravalle F. Pizzoferrato M. Petito V. Lopetuso L. (2018). Skeletal muscle-gut axis: emerging mechanisms of sarcopenia for intestinal and extra intestinal diseases. Minerva Gastroenterol. Dietol. 64, 351–362. 10.23736/S1121-421X.18.02511-4 30016852

[B40] Del Giudice M. Gangestad S. W. (2018). Rethinking IL-6 and CRP: why they are more than inflammatory biomarkers, and why it matters. Brain Behav. Immun. 70, 61–75. 10.1016/j.bbi.2018.02.013 29499302

[B41] Dent E. Morley J. E. Cruz-Jentoft A. J. Arai H. Kritchevsky S. B. Guralnik J. (2018). International clinical practice guidelines for sarcopenia (ICFSR): screening, diagnosis and management. J. Nutr. Health Aging 22, 1148–1161. 10.1007/s12603-018-1139-9 30498820 PMC12280515

[B42] Diao H. Yan F. He Q. Li M. Zheng Q. Zhu Q. (2023). Association between dietary inflammatory index and sarcopenia: a meta-analysis. Nutrients 15, 219. 10.3390/nu15010219 36615879 PMC9824141

[B43] Donini L. M. Busetto L. Bischoff S. C. Cederholm T. Ballesteros-Pomar M. D. Batsis J. A. (2022). Definition and diagnostic criteria for sarcopenic obesity: ESPEN and EASO consensus statement. Obes. Facts 15, 321–335. 10.1159/000521241 35196654 PMC9210010

[B44] Dowery R. Benhamou D. Benchetrit E. Harel O. Nevelsky A. Zisman-Rozen S. (2021). Peripheral B cells repress B-cell regeneration in aging through a TNF-α/IGFBP-1/IGF-1 immune-endocrine axis. Blood 138, 1817–1829. 10.1182/blood.2021012428 34297797 PMC9642783

[B45] Duggal N. A. Pollock R. D. Lazarus N. R. Harridge S. Lord J. M. (2018). Major features of immunesenescence, including reduced thymic output, are ameliorated by high levels of physical activity in adulthood. Aging Cell. 17, e12750. 10.1111/acel.12750 29517845 PMC5847865

[B46] Dumont N. A. Bentzinger C. F. Sincennes M.-C. Rudnicki M. A. (2015). Satellite cells and skeletal muscle regeneration. Compr. Physiol. 5, 1027–1059. 10.1002/cphy.c140068 26140708

[B47] Dupont J. Dedeyne L. Dalle S. Koppo K. Gielen E. (2019). The role of omega-3 in the prevention and treatment of sarcopenia. Aging Clin. Exp. Res. 31, 825–836. 10.1007/s40520-019-01146-1 30784011 PMC6583677

[B48] Dupont J. Antonio L. Dedeyne L. O’Neill T. W. Vanderschueren D. Rastrelli G. (2021). Inflammatory markers are associated with quality of life, physical activity, and gait speed but not sarcopenia in aged men (40-79 years). J. Cachexia Sarcopenia Muscle 12, 1818–1831. 10.1002/jcsm.12785 34523822 PMC8718046

[B49] El Hajj C. Fares S. Chardigny J. M. Boirie Y. Walrand S. (2018). Vitamin D supplementation and muscle strength in pre-sarcopenic elderly Lebanese people: a randomized controlled trial. Arch. Osteoporos. 14, 4. 10.1007/s11657-018-0553-2 30569340

[B50] Engin A. B. (2017). Adipocyte-macrophage cross-talk in obesity. Adv. Exp. Med. Biol. 960, 327–343. 10.1007/978-3-319-48382-5_14 28585206

[B51] Ferrucci L. Fabbri E. (2018). Inflammageing: chronic inflammation in ageing, cardiovascular disease, and frailty. Nat. Rev. Cardiol. 15, 505–522. 10.1038/s41569-018-0064-2 30065258 PMC6146930

[B52] Ferrucci L. Penninx B. W. J. H. Volpato S. Harris T. B. Bandeen-Roche K. Balfour J. (2002). Change in muscle strength explains accelerated decline of physical function in older women with high interleukin-6 serum levels. J. Am. Geriatr. Soc. 50, 1947–1954. 10.1046/j.1532-5415.2002.50605.x 12473005

[B53] Forcina L. Miano C. Musarò A. (2018). The physiopathologic interplay between stem cells and tissue niche in muscle regeneration and the role of IL-6 on muscle homeostasis and diseases. Cytokine Growth Factor Rev. 41, 1–9. 10.1016/j.cytogfr.2018.05.001 29778303

[B54] Fragala M. S. Cadore E. L. Dorgo S. Izquierdo M. Kraemer W. J. Peterson M. D. (2019). Resistance training for older adults: position statement from the national strength and conditioning association. J. Strength Cond. Res. 33, 2019–2052. 10.1519/JSC.0000000000003230 31343601

[B55] Franceschi C. Bonafè M. Valensin S. Olivieri F. De Luca M. Ottaviani E. (2000). Inflamm-aging. An evolutionary perspective on immunosenescence. Ann. N. Y. Acad. Sci. 908, 244–254. 10.1111/j.1749-6632.2000.tb06651.x 10911963

[B56] Franceschi C. Garagnani P. Parini P. Giuliani C. Santoro A. (2018). Inflammaging: a new immune-metabolic viewpoint for age-related diseases. Nat. Rev. Endocrinol. 14, 576–590. 10.1038/s41574-018-0059-4 30046148

[B57] Frasca D. Blomberg B. B. (2020). Adipose tissue, immune aging, and cellular senescence. Semin. Immunopathol. 42, 573–587. 10.1007/s00281-020-00812-1 32785750 PMC7669559

[B58] Fu Y. Nie J.-R. Shang P. Zhang B. Yan D.-W. Hao X. (2024). Tumor necrosis factor α deficiency promotes myogenesis and muscle regeneration. Zool. Res. 45, 951–960. 10.24272/j.issn.2095-8137.2024.039 39021083 PMC11298682

[B59] Fulop T. Larbi A. Dupuis G. Le Page A. Frost E. H. Cohen A. A. (2017). Immunosenescence and inflamm-aging as two sides of the same coin: friends or foes? Front. Immunol. 8, 1960. 10.3389/fimmu.2017.01960 29375577 PMC5767595

[B60] Fulop T. Larbi A. Pawelec G. Khalil A. Cohen A. A. Hirokawa K. (2023). Immunology of aging: the birth of inflammaging. Clin. Rev. Allergy Immunol. 64, 109–122. 10.1007/s12016-021-08899-6 34536213 PMC8449217

[B61] Gaspar R. C. Muñoz V. R. Formigari G. P. Kuga G. K. Nakandakari S. C. B. R. Botezelli J. D. (2018). Acute physical exercise increases the adaptor protein APPL1 in the hypothalamus of Obese mice. Cytokine 110, 87–93. 10.1016/j.cyto.2018.04.013 29705396

[B62] Gianopoulos I. Mantzoros C. S. Daskalopoulou S. S. (2025). Adiponectin and adiponectin receptors in atherosclerosis. Endocr. Rev. 46, 1–25. 10.1210/endrev/bnae021 39106421 PMC11720176

[B63] Gielen E. Beckwée D. Delaere A. De Breucker S. Vandewoude M. Bautmans I. (2021). Nutritional interventions to improve muscle mass, muscle strength, and physical performance in older people: an umbrella review of systematic reviews and meta-analyses. Nutr. Rev. 79, 121–147. 10.1093/nutrit/nuaa011 32483625

[B64] Girousse A. Gil-Ortega M. Bourlier V. Bergeaud C. Sastourné-Arrey Q. Moro C. (2019). The release of adipose stromal cells from subcutaneous adipose tissue regulates ectopic intramuscular adipocyte deposition. Cell. Rep. 27, 323–333.e5. 10.1016/j.celrep.2019.03.038 30970240

[B65] Gomarasca M. Micielska K. Faraldi M. Flis M. Perego S. Banfi G. (2022). Impact of 12-Week moderate-intensity aerobic training on inflammasome complex activation in elderly women. Front. Physiol. 13, 792859. 10.3389/fphys.2022.792859 35273516 PMC8902397

[B66] Goodpaster B. H. Bergman B. C. Brennan A. M. Sparks L. M. (2023). Intermuscular adipose tissue in metabolic disease. Nat. Rev. Endocrinol. 19, 285–298. 10.1038/s41574-022-00784-2 36564490

[B67] Gopinath S. D. Rando T. A. (2008). Stem cell review series: aging of the skeletal muscle stem cell niche. Aging Cell. 7, 590–598. 10.1111/j.1474-9726.2008.00399.x 18462272

[B68] Gray S. R. Mittendorfer B. (2018). Fish oil-derived n-3 polyunsaturated fatty acids for the prevention and treatment of sarcopenia. Curr. Opin. Clin. Nutr. Metab. Care 21, 104–109. 10.1097/MCO.0000000000000441 29232264

[B69] Grosicki G. J. Fielding R. A. Lustgarten M. S. (2018). Gut microbiota contribute to age-related changes in skeletal muscle size, composition, and function: biological basis for a gut-muscle axis. Calcif. Tissue Int. 102, 433–442. 10.1007/s00223-017-0345-5 29058056 PMC5858871

[B70] Grosicki G. J. Barrett B. B. Englund D. A. Liu C. Travison T. G. Cederholm T. (2020). Circulating Interleukin-6 is associated with skeletal muscle strength, quality, and functional adaptation with exercise training in mobility-limited older adults. J. Frailty Aging 9, 57–63. 10.14283/jfa.2019.30 32150215 PMC12275783

[B71] Grun L. K. Maurmann R. M. Scholl J. N. Fogaça M. E. Schmitz C. R. R. Dias C. K. (2023). Obesity drives adipose-derived stem cells into a senescent and dysfunctional phenotype associated with P38MAPK/NF-KB axis. Immun. Ageing 20, 51. 10.1186/s12979-023-00378-0 37821967 PMC10566105

[B72] Guo D. Wang C. Wang Q. Qiao Z. Tang H. (2017). Pantoprazole blocks the JAK2/STAT3 pathway to alleviate skeletal muscle wasting in cancer cachexia by inhibiting inflammatory response. Oncotarget 8, 39640–39648. 10.18632/oncotarget.17387 28489606 PMC5503639

[B73] Hayflick L. Moorhead P. S. (1961). The serial cultivation of human diploid cell strains. Exp. Cell. Res. 25, 585–621. 10.1016/0014-4827(61)90192-6 13905658

[B74] He Y. Xie W. Li H. Jin H. Zhang Y. Li Y. (2021). Cellular senescence in sarcopenia: possible mechanisms and therapeutic potential. Front. Cell. Dev. Biol. 9, 793088. 10.3389/fcell.2021.793088 35083219 PMC8784872

[B75] Heber D. Ingles S. Ashley J. M. Maxwell M. H. Lyons R. F. Elashoff R. M. (1996). Clinical detection of sarcopenic obesity by bioelectrical impedance analysis. Am. J. Clin. Nutr. 64, 472S–477S. 10.1093/ajcn/64.3.472S 8780366

[B76] Hollingworth T. W. Oke S. M. Patel H. Smith T. R. (2021). Getting to grips with sarcopenia: recent advances and practical management for the gastroenterologist. Frontline Gastroenterol. 12, 53–61. 10.1136/flgastro-2019-101348 33489069 PMC7802493

[B77] Hong X. Campanario S. Ramírez-Pardo I. Grima-Terrén M. Isern J. Muñoz-Cánoves P. (2022). Stem cell aging in the skeletal muscle: the importance of communication. Ageing Res. Rev. 73, 101528. 10.1016/j.arr.2021.101528 34818593

[B78] Hood D. A. Memme J. M. Oliveira A. N. Triolo M. (2019). Maintenance of skeletal muscle mitochondria in health, exercise, and aging. Annu. Rev. Physiol. 81, 19–41. 10.1146/annurev-physiol-020518-114310 30216742

[B79] Huang C. Tomata Y. Kakizaki M. Sugawara Y. Hozawa A. Momma H. (2015). High circulating adiponectin levels predict decreased muscle strength among older adults aged 70 years and over: a prospective cohort study. Nutr. Metab. Cardiovasc Dis. 25, 594–601. 10.1016/j.numecd.2015.03.010 25921841

[B80] Huang S.-W. Xu T. Zhang C.-T. Zhou H.-L. (2020). Relationship of peripheral lymphocyte subsets and skeletal muscle mass index in sarcopenia: a cross-sectional study. J. Nutr. Health Aging 24, 325–329. 10.1007/s12603-020-1329-0 32115615 PMC12878528

[B81] Huang W. Hickson L. J. Eirin A. Kirkland J. L. Lerman L. O. (2022). Cellular senescence: the good, the bad and the unknown. Nat. Rev. Nephrol. 18, 611–627. 10.1038/s41581-022-00601-z 35922662 PMC9362342

[B82] Hurst C. Robinson S. M. Witham M. D. Dodds R. M. Granic A. Buckland C. (2022). Resistance exercise as a treatment for sarcopenia: prescription and delivery. Age Ageing 51, afac003. 10.1093/ageing/afac003 35150587 PMC8840798

[B83] Huynh T. Reed C. Blackwell Z. Phelps P. Herrera L. C. P. Almodovar J. (2023). Local IL-10 delivery modulates the immune response and enhances repair of volumetric muscle loss muscle injury. Sci. Rep. 13, 1983. 10.1038/s41598-023-27981-x 36737628 PMC9898301

[B84] Ito S. Nakashima H. Ando K. Kobayashi K. Machino M. Seki T. (2021). Association between low muscle mass and inflammatory cytokines. Biomed. Res. Int. 2021, 5572742. 10.1155/2021/5572742 33997015 PMC8099521

[B85] JafariNasabian P. Inglis J. E. Reilly W. Kelly O. J. Ilich J. Z. (2017). Aging human body: changes in bone, muscle and body fat with consequent changes in nutrient intake. J. Endocrinol. 234, R37–R51. 10.1530/JOE-16-0603 28442508

[B86] James K. Premchand N. Skibinska A. Skibinski G. Nicol M. Mason J. I. (1997). IL-6, DHEA and the ageing process. Mech. Ageing Dev. 93, 15–24. 10.1016/s0047-6374(96)01807-6 9089567

[B87] Kahn C. R. Wang G. Lee K. Y. (2019). Altered adipose tissue and adipocyte function in the pathogenesis of metabolic syndrome. J. Clin. Invest 129, 3990–4000. 10.1172/JCI129187 31573548 PMC6763230

[B88] Kalinkovich A. Livshits G. (2017). Sarcopenic obesity or Obese sarcopenia: a cross talk between age-associated adipose tissue and skeletal muscle inflammation as a main mechanism of the pathogenesis. Ageing Res. Rev. 35, 200–221. 10.1016/j.arr.2016.09.008 27702700

[B89] Kang X. Yang M.-Y. Shi Y.-X. Xie M.-M. Zhu M. Zheng X.-L. (2018). Interleukin-15 facilitates muscle regeneration through modulation of fibro/adipogenic progenitors. Cell. Commun. Signal 16, 42. 10.1186/s12964-018-0251-0 30029643 PMC6053744

[B90] Katzir I. Adler M. Karin O. Mendelsohn-Cohen N. Mayo A. Alon U. (2021). Senescent cells and the incidence of age-related diseases. Aging Cell. 20, e13314. 10.1111/acel.13314 33559235 PMC7963340

[B234] Kaur S. Bansal Y. Kumar R. Bansal G. (2020). A panoramic review of IL-6: Structure, pathophysiological roles and inhibitors. Bioorg. Med. Chem. 28, 115327. 10.1016/j.bmc.2020.115327 31992476

[B91] Kim H. Kim M. Kojima N. Fujino K. Hosoi E. Kobayashi H. (2016). Exercise and nutritional supplementation on community-dwelling elderly Japanese women with sarcopenic obesity: a randomized controlled trial. J. Am. Med. Dir. Assoc. 17, 1011–1019. 10.1016/j.jamda.2016.06.016 27544583

[B92] Kohara K. Ochi M. Tabara Y. Nagai T. Igase M. Miki T. (2011). Leptin in sarcopenic visceral obesity: possible link between adipocytes and myocytes. PLoS One 6, e24633. 10.1371/journal.pone.0024633 21931785 PMC3170390

[B93] Kojta I. Chacińska M. Błachnio-Zabielska A. (2020). Obesity, bioactive lipids, and adipose tissue inflammation in insulin resistance. Nutrients 12, 1305. 10.3390/nu12051305 32375231 PMC7284998

[B94] Komici K. Dello Iacono A. De Luca A. Perrotta F. Bencivenga L. Rengo G. (2021). Adiponectin and sarcopenia: a systematic review with meta-analysis. Front. Endocrinol. (Lausanne) 12, 576619. 10.3389/fendo.2021.576619 33935962 PMC8082154

[B235] Kondo N. Kuroda T. Kobayashi D. (2021). Cytokine networks in the pathogenesis of rheumatoid arthritis. Int. J. Mol. Sci. 22, 10922. 10.3390/ijms222010922 34681582 PMC8539723

[B95] Kraemer W. J. Ratamess N. A. Hymer W. C. Nindl B. C. Fragala M. S. (2020). Growth hormone(s), testosterone, insulin-like growth factors, and cortisol: roles and integration for cellular development and growth with exercise. Front. Endocrinol. (Lausanne) 11, 33. 10.3389/fendo.2020.00033 32158429 PMC7052063

[B96] Krolopp J. E. Thornton S. M. Abbott M. J. (2016). IL-15 activates the Jak3/STAT3 signaling pathway to mediate glucose uptake in skeletal muscle cells. Front. Physiol. 7, 626. 10.3389/fphys.2016.00626 28066259 PMC5167732

[B97] Kunz H. E. Hart C. R. Gries K. J. Parvizi M. Laurenti M. Dalla Man C. (2021). Adipose tissue macrophage populations and inflammation are associated with systemic inflammation and insulin resistance in obesity. Am. J. Physiol. Endocrinol. Metab. 321, E105–E121. 10.1152/ajpendo.00070.2021 33998291 PMC8321823

[B98] Landi F. Marzetti E. Liperoti R. Pahor M. Russo A. Martone A. M. (2013). Nonsteroidal anti-inflammatory drug (NSAID) use and sarcopenia in older people: results from the ilSIRENTE study. J. Am. Med. Dir. Assoc. 14 (626), e9–e13. 10.1016/j.jamda.2013.04.012 23747142

[B99] LeDrew M. Sadri P. Peil A. Farahnak Z. (2025). Muscle biomarkers as molecular signatures for early detection and monitoring of muscle health in aging. Nutrients 17, 2758. 10.3390/nu17172758 40944148 PMC12430472

[B100] Leduc-Gaudet J.-P. Hussain S. N. A. Barreiro E. Gouspillou G. (2021). Mitochondrial dynamics and mitophagy in skeletal muscle health and aging. Int. J. Mol. Sci. 22, 8179. 10.3390/ijms22158179 34360946 PMC8348122

[B101] Li P. Chang M. (2021). Roles of PRR-mediated signaling pathways in the regulation of oxidative stress and inflammatory diseases. Int. J. Mol. Sci. 22, 7688. 10.3390/ijms22147688 34299310 PMC8306625

[B102] Li C.-W. Yu K. Shyh-Chang N. Li G.-X. Jiang L.-J. Yu S.-L. (2019). Circulating factors associated with sarcopenia during ageing and after intensive lifestyle intervention. J. Cachexia Sarcopenia Muscle 10, 586–600. 10.1002/jcsm.12417 30969486 PMC6596393

[B103] Li C.-W. Yu K. Shyh-Chang N. Li G.-X. Yu S.-L. Liu H.-J. (2021). Sterol metabolism and protein metabolism are differentially correlated with sarcopenia in Asian Chinese men and women. Cell. Prolif. 54, e12989. 10.1111/cpr.12989 33609051 PMC8016649

[B104] Li C.-W. Yu K. Shyh-Chang N. Jiang Z. Liu T. Ma S. (2022). Pathogenesis of sarcopenia and the relationship with fat mass: descriptive review. J. Cachexia Sarcopenia Muscle 13, 781–794. 10.1002/jcsm.12901 35106971 PMC8977978

[B105] Ligthart-Melis G. C. Luiking Y. C. Kakourou A. Cederholm T. Maier A. B. de van der Schueren M. A. E. (2020). Frailty, sarcopenia, and malnutrition frequently (Co-)occur in hospitalized older adults: a systematic review and meta-analysis. J. Am. Med. Dir. Assoc. 21, 1216–1228. 10.1016/j.jamda.2020.03.006 32327302

[B106] Liu H.-W. Chang S.-J. (2018). Moderate exercise suppresses NF-κB signaling and activates the SIRT1-AMPK-PGC1α axis to attenuate muscle loss in diabetic Db/Db mice. Front. Physiol. 9, 636. 10.3389/fphys.2018.00636 29896118 PMC5987703

[B107] Liu G. Y. Sabatini D. M. (2020). mTOR at the nexus of nutrition, growth, ageing and disease. Nat. Rev. Mol. Cell. Biol. 21, 183–203. 10.1038/s41580-019-0199-y 31937935 PMC7102936

[B108] Liu C. Cheung W.-H. Li J. Chow S. K.-H. Yu J. Wong S. H. (2021a). Understanding the gut microbiota and sarcopenia: a systematic review. J. Cachexia Sarcopenia Muscle 12, 1393–1407. 10.1002/jcsm.12784 34523250 PMC8718038

[B109] Liu H.-C. Han D.-S. Hsu C.-C. Wang J.-S. (2021b). Circulating MicroRNA-486 and MicroRNA-146a serve as potential biomarkers of sarcopenia in the older adults. BMC Geriatr. 21, 86. 10.1186/s12877-021-02040-0 33516190 PMC7847166

[B110] Liu J. Zhang X. Cheng Y. Cao X. (2021c). Dendritic cell migration in inflammation and immunity. Cell. Mol. Immunol. 18, 2461–2471. 10.1038/s41423-021-00726-4 34302064 PMC8298985

[B111] Liu G. Jiang S. Xie W. Liu X. Yang G. Lu W. (2025a). Biomarkers for sarcopenia, muscle mass, muscle strength, and physical performance: an umbrella review. J. Transl. Med. 23, 650. 10.1186/s12967-025-06575-3 40506715 PMC12160432

[B112] Liu J. Niu D. Tang Y. Zheng R. Qin Y. Cheng X. (2025b). Beta-hydroxy-beta-methylbutyrate (HMB) ameliorates DSS-Induced colitis by inhibiting ERK/NF-κB activation in macrophages. Phytomedicine 139, 156492. 10.1016/j.phymed.2025.156492 39978274

[B113] Lopes L. C. C. Mota J. F. Prestes J. Schincaglia R. M. Silva D. M. Queiroz N. P. (2019). Intradialytic resistance training improves functional capacity and lean mass gain in individuals on hemodialysis: a randomized pilot trial. Arch. Phys. Med. Rehabil. 100, 2151–2158. 10.1016/j.apmr.2019.06.006 31278924

[B114] Lu Y. Lim W. S. Jin X. Zin Nyunt M. S. Fulop T. Gao Q. (2022). Lower insulin level is associated with sarcopenia in community-dwelling frail and non-frail older adults. Front. Med. (Lausanne) 9, 971622. 10.3389/fmed.2022.971622 36482911 PMC9722960

[B115] Luo Y. Liu M. (2016). Adiponectin: a versatile player of innate immunity. J. Mol. Cell. Biol. 8, 120–128. 10.1093/jmcb/mjw012 26993045 PMC4816149

[B116] Lynch G. M. Murphy C. H. Castro E. de M. Roche H. M. (2020). Inflammation and metabolism: the role of adiposity in sarcopenic obesity. Proc. Nutr. Soc., 1–13. 10.1017/S0029665120007119 32669148

[B117] Mankhong S. Kim S. Moon S. Kwak H.-B. Park D.-H. Kang J.-H. (2020). Experimental models of sarcopenia: bridging molecular mechanism and therapeutic strategy. Cells 9, 1385. 10.3390/cells9061385 32498474 PMC7348939

[B118] Marcangeli V. Youssef L. Dulac M. Carvalho L. P. Hajj-Boutros G. Reynaud O. (2022). Impact of high-intensity interval training with or without l-citrulline on physical performance, skeletal muscle, and adipose tissue in Obese older adults. J. Cachexia Sarcopenia Muscle 13, 1526–1540. 10.1002/jcsm.12955 35257499 PMC9178162

[B119] Marchi S. Guilbaud E. Tait S. W. G. Yamazaki T. Galluzzi L. (2023). Mitochondrial control of inflammation. Nat. Rev. Immunol. 23, 159–173. 10.1038/s41577-022-00760-x 35879417 PMC9310369

[B120] Martínez-Arnau F. M. Fonfría-Vivas R. Buigues C. Castillo Y. Molina P. Hoogland A. J. (2020). Effects of leucine administration in sarcopenia: a randomized and placebo-controlled clinical trial. Nutrients 12, 932. 10.3390/nu12040932 32230954 PMC7230494

[B121] Meiliana A. Dewi N. M. Defi I. R. Rosdianto A. M. Qiantori A. A. Wijaya A. (2024). Sarcopenic obesity: the underlying molecular pathophysiology and prospect therapies. Indones. Biomed. J. 16, 292–308. 10.18585/inabj.v16i4.3176

[B122] Midttun M. Overgaard K. Zerahn B. Pedersen M. Rashid A. Østergren P. B. (2024). Beneficial effects of exercise, testosterone, vitamin D, calcium and protein in older men-A randomized clinical trial. J. Cachexia Sarcopenia Muscle 15, 1451–1462. 10.1002/jcsm.13498 38890228 PMC11294024

[B123] Mikó A. Pótó L. Mátrai P. Hegyi P. Füredi N. Garami A. (2018). Gender difference in the effects of interleukin-6 on grip strength - a systematic review and meta-analysis. BMC Geriatr. 18, 107. 10.1186/s12877-018-0798-z 29739343 PMC5941705

[B124] Minihane A. M. Vinoy S. Russell W. R. Baka A. Roche H. M. Tuohy K. M. (2015). Low-grade inflammation, diet composition and health: current research evidence and its translation. Br. J. Nutr. 114, 999–1012. 10.1017/S0007114515002093 26228057 PMC4579563

[B125] Mohan D. Rossiter H. Watz H. Fogarty C. Evans R. A. Man W. (2023). Selective androgen receptor modulation for muscle weakness in chronic obstructive pulmonary disease: a randomised control trial. Thorax 78, 258–266. 10.1136/thorax-2021-218360 36283827 PMC9985744

[B126] Mori H. Tokuda Y. (2018). Effect of whey protein supplementation after resistance exercise on the muscle mass and physical function of healthy older women: a randomized controlled trial. Geriatr. Gerontol. Int. 18, 1398–1404. 10.1111/ggi.13499 30113122

[B127] Mourkioti F. Kratsios P. Luedde T. Song Y.-H. Delafontaine P. Adami R. (2006). Targeted ablation of IKK2 improves skeletal muscle strength, maintains mass, and promotes regeneration. J. Clin. Invest 116, 2945–2954. 10.1172/JCI28721 17080195 PMC1626136

[B128] Mouton A. J. Li X. Hall M. E. Hall J. E. (2020). Obesity, hypertension, and cardiac dysfunction: novel roles of immunometabolism in macrophage activation and inflammation. Circ. Res. 126, 789–806. 10.1161/CIRCRESAHA.119.312321 32163341 PMC7255054

[B129] Murakami T. Inagaki N. Kondoh H. (2022). Cellular senescence in diabetes mellitus: distinct senotherapeutic strategies for adipose tissue and pancreatic β cells. Front. Endocrinol. (Lausanne) 13, 869414. 10.3389/fendo.2022.869414 35432205 PMC9009089

[B130] Nabuco H. C. G. Tomeleri C. M. Fernandes R. R. Sugihara Junior P. Cavalcante E. F. Cunha P. M. (2019). Effect of whey protein supplementation combined with resistance training on body composition, muscular strength, functional capacity, and plasma-metabolism biomarkers in older women with sarcopenic obesity: a randomized, double-blind, placebo-controlled trial. Clin. Nutr. ESPEN 32, 88–95. 10.1016/j.clnesp.2019.04.007 31221297

[B131] Nagata K. Nishiyama C. (2021). IL-10 in mast cell-mediated immune responses: anti-inflammatory and proinflammatory roles. Int. J. Mol. Sci. 22, 4972. 10.3390/ijms22094972 34067047 PMC8124430

[B132] Namioka N. Hanyu H. Hirose D. Hatanaka H. Sato T. Shimizu S. (2017). Oxidative stress and inflammation are associated with physical frailty in patients with Alzheimer’s disease. Geriatr. Gerontol. Int. 17, 913–918. 10.1111/ggi.12804 27296166

[B133] Nguyen T. T. Corvera S. (2024). Adipose tissue as a linchpin of organismal ageing. Nat. Metab. 6, 793–807. 10.1038/s42255-024-01046-3 38783156 PMC11238912

[B134] Ou M.-Y. Zhang H. Tan P.-C. Zhou S.-B. Li Q.-F. (2022). Adipose tissue aging: mechanisms and therapeutic implications. Cell. Death Dis. 13, 300. 10.1038/s41419-022-04752-6 35379822 PMC8980023

[B135] Ouyang W. O’Garra A. (2019). IL-10 family cytokines IL-10 and IL-22: from basic science to clinical translation. Immunity 50, 871–891. 10.1016/j.immuni.2019.03.020 30995504

[B136] Özcan S. Alessio N. Acar M. B. Mert E. Omerli F. Peluso G. (2016). Unbiased analysis of senescence associated secretory phenotype (SASP) to identify common components following different genotoxic stresses. Aging (Albany NY) 8, 1316–1329. 10.18632/aging.100971 27288264 PMC4993333

[B137] O’Leary M. F. Wallace G. R. Bennett A. J. Tsintzas K. Jones S. W. (2017). IL-15 promotes human myogenesis and mitigates the detrimental effects of TNFα on myotube development. Sci. Rep. 7, 12997. 10.1038/s41598-017-13479-w 29021612 PMC5636823

[B138] Palla A. R. Ravichandran M. Wang Y. X. Alexandrova L. Yang A. V. Kraft P. (2021). Inhibition of prostaglandin-degrading enzyme 15-PGDH rejuvenates aged muscle mass and strength. Science 371, eabc8059. 10.1126/science.abc8059 33303683 PMC7938328

[B139] Paolucci E. M. Loukov D. Bowdish D. M. E. Heisz J. J. (2018). Exercise reduces depression and inflammation but intensity matters. Biol. Psychol. 133, 79–84. 10.1016/j.biopsycho.2018.01.015 29408464

[B140] Paris M. T. Bell K. E. Mourtzakis M. (2020). Myokines and adipokines in sarcopenia: understanding cross-talk between skeletal muscle and adipose tissue and the role of exercise. Curr. Opin. Pharmacol. 52, 61–66. 10.1016/j.coph.2020.06.003 32668398

[B141] Park Y. Choi J.-E. Hwang H.-S. (2018). Protein supplementation improves muscle mass and physical performance in undernourished prefrail and frail elderly subjects: a randomized, double-blind, placebo-controlled trial. Am. J. Clin. Nutr. 108, 1026–1033. 10.1093/ajcn/nqy214 30475969

[B142] Pascual-Fernández J. Fernández-Montero A. Córdova-Martínez A. Pastor D. Martínez-Rodríguez A. Roche E. (2020). Sarcopenia: molecular pathways and potential targets for intervention. Int. J. Mol. Sci. 21, 8844. 10.3390/ijms21228844 33266508 PMC7700275

[B143] Peoples J. N. Saraf A. Ghazal N. Pham T. T. Kwong J. Q. (2019). Mitochondrial dysfunction and oxidative stress in heart disease. Exp. Mol. Med. 51, 1–13. 10.1038/s12276-019-0355-7 31857574 PMC6923355

[B144] Pérez-Pérez A. Sánchez-Jiménez F. Vilariño-García T. Sánchez-Margalet V. (2020). Role of leptin in inflammation and *vice versa* . Int. J. Mol. Sci. 21, 5887. 10.3390/ijms21165887 32824322 PMC7460646

[B145] Petermann-Rocha F. Balntzi V. Gray S. R. Lara J. Ho F. K. Pell J. P. (2022). Global prevalence of sarcopenia and severe sarcopenia: a systematic review and meta-analysis. J. Cachexia Sarcopenia Muscle 13, 86–99. 10.1002/jcsm.12783 34816624 PMC8818604

[B146] Picca A. Lezza A. M. S. Leeuwenburgh C. Pesce V. Calvani R. Landi F. (2017). Fueling inflamm-aging through mitochondrial dysfunction: mechanisms and molecular targets. Int. J. Mol. Sci. 18, 933. 10.3390/ijms18050933 28452964 PMC5454846

[B147] Pistilli E. E. Quinn L. S. (2013). From anabolic to oxidative: reconsidering the roles of IL-15 and IL-15Rα in skeletal muscle. Exerc Sport Sci. Rev. 41, 100–106. 10.1097/JES.0b013e318275d230 23072822 PMC5317349

[B148] Ponnusamy S. Sullivan R. D. You D. Zafar N. He Yang C. Thiyagarajan T. (2017). Androgen receptor agonists increase lean mass, improve cardiopulmonary functions and extend survival in preclinical models of Duchenne muscular dystrophy. Hum. Mol. Genet. 26, 2526–2540. 10.1093/hmg/ddx150 28453658 PMC6251687

[B149] Pope J. E. Choy E. H. (2021). C-reactive protein and implications in rheumatoid arthritis and associated comorbidities. Semin. Arthritis Rheum. 51, 219–229. 10.1016/j.semarthrit.2020.11.005 33385862

[B150] Potes Y. de Luxán-Delgado B. Rodriguez-González S. Guimarães M. R. M. Solano J. J. Fernández-Fernández M. (2017). Overweight in elderly people induces impaired autophagy in skeletal muscle. Free Radic. Biol. Med. 110, 31–41. 10.1016/j.freeradbiomed.2017.05.018 28549989

[B151] Pototschnig I. Feiler U. Diwoky C. Vesely P. W. Rauchenwald T. Paar M. (2023). Interleukin-6 initiates muscle- and adipose tissue wasting in a novel C57BL/6 model of cancer-associated cachexia. J. Cachexia Sarcopenia Muscle 14, 93–107. 10.1002/jcsm.13109 36351437 PMC9891934

[B152] Poulsen N. B. Lambert M. N. T. Jeppesen P. B. (2020). The effect of plant derived bioactive compounds on inflammation: a systematic review and meta-analysis. Mol. Nutr. Food Res. 64, e2000473. 10.1002/mnfr.202000473 32761736

[B153] Prado C. M. Batsis J. A. Donini L. M. Gonzalez M. C. Siervo M. (2024). Sarcopenic obesity in older adults: a clinical overview. Nat. Rev. Endocrinol. 20, 261–277. 10.1038/s41574-023-00943-z 38321142 PMC12854800

[B154] Prokopidis K. Cervo M. M. Gandham A. Scott D. (2020). Impact of protein intake in older adults with sarcopenia and obesity: a gut microbiota perspective. Nutrients 12, 2285. 10.3390/nu12082285 32751533 PMC7468805

[B155] Puzianowska-Kuźnicka M. Owczarz M. Wieczorowska-Tobis K. Nadrowski P. Chudek J. Slusarczyk P. (2016). Interleukin-6 and C-reactive protein, successful aging, and mortality: the PolSenior study. Immun. Ageing 13, 21. 10.1186/s12979-016-0076-x 27274758 PMC4891873

[B156] Qaseem A. Horwitch C. A. Vijan S. Etxeandia-Ikobaltzeta I. Kansagara D. Clinical Guidelines Committee of the American College of Physicians (2020). Testosterone treatment in adult men with age-related low testosterone: a clinical guideline from the American college of physicians. Ann. Intern Med. 172, 126–133. 10.7326/M19-0882 31905405

[B157] Rahman M. M. Bhattacharya A. Banu J. Fernandes G. (2007). Conjugated linoleic acid protects against age-associated bone loss in C57BL/6 female mice. J. Nutr. Biochem. 18, 467–474. 10.1016/j.jnutbio.2006.08.002 16997541

[B158] Reidy P. T. Lindsay C. C. McKenzie A. I. Fry C. S. Supiano M. A. Marcus R. L. (2018). Aging-related effects of bed rest followed by eccentric exercise rehabilitation on skeletal muscle macrophages and insulin sensitivity. Exp. Gerontol. 107, 37–49. 10.1016/j.exger.2017.07.001 28705613 PMC5762440

[B159] Reyes-Farias M. Fos-Domenech J. Serra D. Herrero L. Sánchez-Infantes D. (2021). White adipose tissue dysfunction in obesity and aging. Biochem. Pharmacol. 192, 114723. 10.1016/j.bcp.2021.114723 34364887

[B160] Rizo-Téllez S. A. Sekheri M. Filep J. G. (2023). C-reactive protein: a target for therapy to reduce inflammation. Front. Immunol. 14, 1237729. 10.3389/fimmu.2023.1237729 37564640 PMC10410079

[B161] Rodriguez-Lopez C. Alcazar J. Sanchez-Martin C. Baltasar-Fernandez I. Ara I. Csapo R. (2022). Neuromuscular adaptations after 12 weeks of light-vs. heavy-load power-oriented resistance training in older adults. Scand. J. Med. Sci. Sports 32, 324–337. 10.1111/sms.14073 34618979

[B162] Rolland Y. Dray C. Vellas B. Barreto P. D. S. (2023). Current and investigational medications for the treatment of sarcopenia. Metabolism 149, 155597. 10.1016/j.metabol.2023.155597 37348598

[B163] Rong Y.-D. Bian A.-L. Hu H.-Y. Ma Y. Zhou X.-Z. (2018). Study on relationship between elderly sarcopenia and inflammatory cytokine IL-6, anti-inflammatory cytokine IL-10. BMC Geriatr. 18, 308. 10.1186/s12877-018-1007-9 30541467 PMC6292155

[B164] Rose G. L. Skinner T. L. Mielke G. I. Schaumberg M. A. (2021). The effect of exercise intensity on chronic inflammation: a systematic review and meta-analysis. J. Sci. Med. Sport 24, 345–351. 10.1016/j.jsams.2020.10.004 33153926

[B165] Rosenberg I. H. (1997). Sarcopenia: origins and clinical relevance. J. Nutr. 127, 990S–991S. 10.1093/jn/127.5.990S 9164280

[B166] Russell A. P. Wallace M. A. Kalanon M. Zacharewicz E. Della Gatta P. A. Garnham A. (2017). Striated muscle activator of rho signalling (STARS) is reduced in ageing human skeletal muscle and targeted by miR-628-5p. Acta Physiol. (Oxf) 220, 263–274. 10.1111/apha.12819 27739650

[B167] Sadjapong U. Yodkeeree S. Sungkarat S. Siviroj P. (2020). Multicomponent exercise Program reduces frailty and inflammatory biomarkers and improves physical performance in community-dwelling older adults: a randomized controlled trial. Int. J. Environ. Res. Public Health 17, 3760. 10.3390/ijerph17113760 32466446 PMC7312630

[B168] Saini J. McPhee J. S. Al-Dabbagh S. Stewart C. E. Al-Shanti N. (2016). Regenerative function of immune system: modulation of muscle stem cells. Ageing Res. Rev. 27, 67–76. 10.1016/j.arr.2016.03.006 27039885

[B169] Sáinz N. Rodríguez A. Catalán V. Becerril S. Ramírez B. Gómez-Ambrosi J. (2009). Leptin administration favors muscle mass accretion by decreasing FoxO3a and increasing PGC-1alpha in ob/ob mice. PLoS One 4, e6808. 10.1371/journal.pone.0006808 19730740 PMC2733298

[B170] Sakers A. De Siqueira M. K. Seale P. Villanueva C. J. (2022). Adipose-tissue plasticity in health and disease. Cell. 185, 419–446. 10.1016/j.cell.2021.12.016 35120662 PMC11152570

[B171] Sánchez-Sánchez J. L. Mañas A. García-García F. J. Ara I. Carnicero J. A. Walter S. (2019). Sedentary behaviour, physical activity, and sarcopenia among older adults in the TSHA: isotemporal substitution model. J. Cachexia Sarcopenia Muscle 10, 188–198. 10.1002/jcsm.12369 30920779 PMC6438335

[B172] Santoro A. Bientinesi E. Monti D. (2021). Immunosenescence and inflammaging in the aging process: age-related diseases or longevity? Ageing Res. Rev. 71, 101422. 10.1016/j.arr.2021.101422 34391943

[B173] Sardeli A. V. Tomeleri C. M. Cyrino E. S. Fernhall B. Cavaglieri C. R. Chacon-Mikahil M. P. T. (2018). Effect of resistance training on inflammatory markers of older adults: a meta-analysis. Exp. Gerontol. 111, 188–196. 10.1016/j.exger.2018.07.021 30071283

[B174] Sciorati C. Gamberale R. Monno A. Citterio L. Lanzani C. De Lorenzo R. (2020). Pharmacological blockade of TNFα prevents sarcopenia and prolongs survival in aging mice. Aging (Albany NY) 12, 23497–23508. 10.18632/aging.202200 33260150 PMC7762456

[B175] Severinsen M. C. K. Pedersen B. K. (2020). Muscle-organ crosstalk: the emerging roles of myokines. Endocr. Rev. 41, 594–609. 10.1210/endrev/bnaa016 32393961 PMC7288608

[B176] Shen Y. Shi Q. Nong K. Li S. Yue J. Huang J. (2023). Exercise for sarcopenia in older people: a systematic review and network meta-analysis. J. Cachexia Sarcopenia Muscle 14, 1199–1211. 10.1002/jcsm.13225 37057640 PMC10235889

[B177] Shi J. Bei Y. Kong X. Liu X. Lei Z. Xu T. (2017). miR-17-3p contributes to exercise-induced cardiac growth and protects against myocardial ischemia-reperfusion injury. Theranostics 7, 664–676. 10.7150/thno.15162 28255358 PMC5327641

[B178] Shin M. J. Jeon Y. K. Kim I. J. (2018). Testosterone and sarcopenia. World J. Mens. Health 36, 192–198. 10.5534/wjmh.180001 29756416 PMC6119844

[B179] Shin M. S. Yim K. Moon K. Park H.-J. Mohanty S. Kim J. W. (2019). Dissecting alterations in human CD8+ T cells with aging by high-dimensional single cell mass cytometry. Clin. Immunol. 200, 24–30. 10.1016/j.clim.2019.01.005 30659916 PMC6443094

[B180] Shivappa N. Steck S. E. Hurley T. G. Hussey J. R. Ma Y. Ockene I. S. (2014). A population-based dietary inflammatory index predicts levels of C-reactive protein in the seasonal variation of blood cholesterol study (SEASONS). Public Health Nutr. 17, 1825–1833. 10.1017/S1368980013002565 24107546 PMC3983179

[B181] Shokri-Mashhadi N. Moradi S. Heidari Z. Saadat S. (2021). Association of circulating C-reactive protein and high-sensitivity C-reactive protein with components of sarcopenia: a systematic review and meta-analysis of observational studies. Exp. Gerontol. 150, 111330. 10.1016/j.exger.2021.111330 33848566

[B182] Smith G. I. Julliand S. Reeds D. N. Sinacore D. R. Klein S. Mittendorfer B. (2015). Fish oil-derived n-3 PUFA therapy increases muscle mass and function in healthy older adults. Am. J. Clin. Nutr. 102, 115–122. 10.3945/ajcn.114.105833 25994567 PMC4480667

[B183] Snyder P. J. Bhasin S. Cunningham G. R. Matsumoto A. M. Stephens-Shields A. J. Cauley J. A. (2016). Effects of testosterone treatment in older men. N. Engl. J. Med. 374, 611–624. 10.1056/NEJMoa1506119 26886521 PMC5209754

[B184] Snyder P. J. Bauer D. C. Ellenberg S. S. Cauley J. A. Buhr K. A. Bhasin S. (2024). Testosterone treatment and fractures in men with hypogonadism. N. Engl. J. Med. 390, 203–211. 10.1056/NEJMoa2308836 38231621

[B185] Sproston N. R. Ashworth J. J. (2018). Role of C-Reactive protein at sites of inflammation and infection. Front. Immunol. 9, 754. 10.3389/fimmu.2018.00754 29706967 PMC5908901

[B186] Storer T. W. Basaria S. Traustadottir T. Harman S. M. Pencina K. Li Z. (2017). Effects of testosterone supplementation for 3 years on muscle performance and physical function in older men. J. Clin. Endocrinol. Metab. 102, 583–593. 10.1210/jc.2016-2771 27754805 PMC5413164

[B187] Stout M. B. Justice J. N. Nicklas B. J. Kirkland J. L. (2017). Physiological aging: links among adipose tissue dysfunction, diabetes, and frailty. Physiol. (Bethesda) 32, 9–19. 10.1152/physiol.00012.2016 27927801 PMC5338596

[B188] Subramaniam K. Fallon K. Ruut T. Lane D. McKay R. Shadbolt B. (2015). Infliximab reverses inflammatory muscle wasting (Sarcopenia) in Crohn’s disease. Aliment. Pharmacol. Ther. 41, 419–428. 10.1111/apt.13058 25580985

[B189] Sumi K. Ashida K. Nakazato K. (2020). Resistance exercise with anti-inflammatory foods attenuates skeletal muscle atrophy induced by chronic inflammation. J. Appl. Physiol. 1985 (128), 197–211. 10.1152/japplphysiol.00585.2019 31804892

[B190] Suryadevara V. Hudgins A. D. Rajesh A. Pappalardo A. Karpova A. Dey A. K. (2024). SenNet recommendations for detecting senescent cells in different tissues. Nat. Rev. Mol. Cell. Biol. 25, 1001–1023. 10.1038/s41580-024-00738-8 38831121 PMC11578798

[B191] Taekema D. G. Westendorp R. G. J. Frölich M. Gussekloo J. (2007). High innate production capacity of tumor necrosis factor-alpha and decline of handgrip strength in old age. Mech. Ageing Dev. 128, 517–521. 10.1016/j.mad.2007.07.001 17714763

[B192] Tagliaferri C. Wittrant Y. Davicco M.-J. Walrand S. Coxam V. (2015). Muscle and bone, two interconnected tissues. Ageing Res. Rev. 21, 55–70. 10.1016/j.arr.2015.03.002 25804855

[B233] Tanaka T. Narazaki M. Kishimoto T. (2018). Interleukin (IL-6) Immunotherapy. Cold Spring Harb. Perspect Biol. 10, a028456. 10.1101/cshperspect.a028456 28778870 PMC6071487

[B193] Tang H. Inoki K. Brooks S. V. Okazawa H. Lee M. Wang J. (2019). mTORC1 underlies age-related muscle fiber damage and loss by inducing oxidative stress and catabolism. Aging Cell. 18, e12943. 10.1111/acel.12943 30924297 PMC6516169

[B194] Tanigaki K. Chambliss K. L. Yuhanna I. S. Sacharidou A. Ahmed M. Atochin D. N. (2016). Endothelial Fcγ receptor IIB activation blunts insulin delivery to skeletal muscle to cause insulin resistance in mice. Diabetes 65, 1996–2005. 10.2337/db15-1605 27207525 PMC4915578

[B195] Thevaranjan N. Puchta A. Schulz C. Naidoo A. Szamosi J. C. Verschoor C. P. (2017). Age-associated microbial dysbiosis promotes intestinal permeability, systemic inflammation, and macrophage dysfunction. Cell. Host Microbe 21, 455–466.e4. 10.1016/j.chom.2017.03.002 28407483 PMC5392495

[B196] Thoma A. Lightfoot A. P. (2018). NF-kB and inflammatory cytokine signalling: role in skeletal muscle atrophy. Adv. Exp. Med. Biol. 1088, 267–279. 10.1007/978-981-13-1435-3_12 30390256

[B197] Thomas R. Wang W. Su D.-M. (2020). Contributions of age-related thymic involution to immunosenescence and inflammaging. Immun. Ageing 17, 2. 10.1186/s12979-020-0173-8 31988649 PMC6971920

[B198] Ticinesi A. Lauretani F. Milani C. Nouvenne A. Tana C. Del Rio D. (2017). Aging gut microbiota at the cross-road between nutrition, physical frailty, and sarcopenia: is there a gut-muscle axis? Nutrients 9, 1303. 10.3390/nu9121303 29189738 PMC5748753

[B199] Ting A. T. Bertrand M. J. M. (2016). More to life than NF-κB in TNFR1 signaling. Trends Immunol. 37, 535–545. 10.1016/j.it.2016.06.002 27424290 PMC5076853

[B200] Tournadre A. Pereira B. Dutheil F. Giraud C. Courteix D. Sapin V. (2017). Changes in body composition and metabolic profile during interleukin 6 inhibition in rheumatoid arthritis. J. Cachexia Sarcopenia Muscle 8, 639–646. 10.1002/jcsm.12189 28316139 PMC5566648

[B201] Troesch B. Eggersdorfer M. Laviano A. Rolland Y. Smith A. D. Warnke I. (2020). Expert opinion on benefits of long-chain Omega-3 fatty acids (DHA and EPA) in aging and clinical nutrition. Nutrients 12, 2555. 10.3390/nu12092555 32846900 PMC7551800

[B202] Tuttle C. S. L. Thang L. A. N. Maier A. B. (2020). Markers of inflammation and their association with muscle strength and mass: a systematic review and meta-analysis. Ageing Res. Rev. 64, 101185. 10.1016/j.arr.2020.101185 32992047

[B203] Uchitomi R. Oyabu M. Kamei Y. (2020). Vitamin D and sarcopenia: potential of vitamin D supplementation in sarcopenia prevention and treatment. Nutrients 12, 3189. 10.3390/nu12103189 33086536 PMC7603112

[B204] van Loo G. Bertrand M. J. M. (2023). Death by TNF: a road to inflammation. Nat. Rev. Immunol. 23, 289–303. 10.1038/s41577-022-00792-3 36380021 PMC9665039

[B205] Vignali J. D. Pak K. C. Beverley H. R. DeLuca J. P. Downs J. W. Kress A. T. (2023). Systematic review of safety of selective androgen receptor modulators in healthy adults: implications for recreational users. J. Xenobiot. 13, 218–236. 10.3390/jox13020017 37218811 PMC10204391

[B206] Virtanen J. K. Mursu J. Voutilainen S. Tuomainen T.-P. (2018). The associations of serum n-6 polyunsaturated fatty acids with serum C-reactive protein in men: the kuopio ischaemic heart disease risk factor study. Eur. J. Clin. Nutr. 72, 342–348. 10.1038/s41430-017-0009-6 29515239

[B207] Voss S. C. Nikolovski Z. Bourdon P. C. Alsayrafi M. Schumacher Y. O. (2016). The effect of cumulative endurance exercise on leptin and adiponectin and their role as markers to monitor training load. Biol. Sport 33, 23–28. 10.5604/20831862.1180173 26985130 PMC4786583

[B208] Wåhlin-Larsson B. Wilkinson D. J. Strandberg E. Hosford-Donovan A. Atherton P. J. Kadi F. (2017). Mechanistic links underlying the impact of C-Reactive protein on muscle mass in elderly. Cell. Physiol. Biochem. 44, 267–278. 10.1159/000484679 29130969

[B209] Wang T. (2022). Searching for the link between inflammaging and sarcopenia. Ageing Res. Rev. 77, 101611. 10.1016/j.arr.2022.101611 35307560

[B210] Wang Y. Welc S. S. Wehling-Henricks M. Tidball J. G. (2018). Myeloid cell-derived tumor necrosis factor-alpha promotes sarcopenia and regulates muscle cell fusion with aging muscle fibers. Aging Cell. 17, e12828. 10.1111/acel.12828 30256507 PMC6260911

[B211] Wang Y. Wehling-Henricks M. Welc S. S. Fisher A. L. Zuo Q. Tidball J. G. (2019). Aging of the immune system causes reductions in muscle stem cell populations, promotes their shift to a fibrogenic phenotype, and modulates sarcopenia. FASEB J. 33, 1415–1427. 10.1096/fj.201800973R 30130434 PMC6355087

[B212] Wang L. Hong W. Zhu H. He Q. Yang B. Wang J. (2024). Macrophage senescence in health and diseases. Acta Pharm. Sin. B 14, 1508–1524. 10.1016/j.apsb.2024.01.008 38572110 PMC10985037

[B213] Wei S. Nguyen T. T. Zhang Y. Ryu D. Gariani K. (2023). Sarcopenic obesity: epidemiology, pathophysiology, cardiovascular disease, mortality, and management. Front. Endocrinol. (Lausanne) 14, 1185221. 10.3389/fendo.2023.1185221 37455897 PMC10344359

[B214] Welc S. S. Wehling-Henricks M. Antoun J. Ha T. T. Tous I. Tidball J. G. (2020). Differential effects of myeloid cell PPARδ and IL-10 in regulating macrophage recruitment, phenotype, and regeneration following acute muscle injury. J. Immunol. 205, 1664–1677. 10.4049/jimmunol.2000247 32817369 PMC7484367

[B215] Wu J. Lin S. Chen W. Lian G. Wu W. Chen A. (2023). TNF-α contributes to sarcopenia through caspase-8/caspase-3/GSDME-mediated pyroptosis. Cell. Death Discov. 9, 76. 10.1038/s41420-023-01365-6 36823174 PMC9950087

[B216] Xia Z. Cholewa J. Zhao Y. Shang H.-Y. Yang Y.-Q. Araújo Pessôa K. (2017). Targeting inflammation and downstream protein metabolism in sarcopenia: a brief Up-Dated description of concurrent exercise and leucine-based multimodal intervention. Front. Physiol. 8, 434. 10.3389/fphys.2017.00434 28690550 PMC5479895

[B217] Xian H. Watari K. Sanchez-Lopez E. Offenberger J. Onyuru J. Sampath H. (2022). Oxidized DNA fragments exit mitochondria *via* mPTP- and VDAC-Dependent channels to activate NLRP3 inflammasome and interferon signaling. Immunity 55, 1370–1385.e8. 10.1016/j.immuni.2022.06.007 35835107 PMC9378606

[B218] Xiang Y. Dai J. Xu L. Li X. Jiang J. Xu J. (2021). Research progress in immune microenvironment regulation of muscle atrophy induced by peripheral nerve injury. Life Sci. 287, 120117. 10.1016/j.lfs.2021.120117 34740577

[B219] Xu M. Tchkonia T. Ding H. Ogrodnik M. Lubbers E. R. Pirtskhalava T. (2015). JAK inhibition alleviates the cellular senescence-associated secretory phenotype and frailty in old age. Proc. Natl. Acad. Sci. U. S. A. 112, E6301–E6310. 10.1073/pnas.1515386112 26578790 PMC4655580

[B220] Xu H. Ranjit R. Richardson A. Van Remmen H. (2021). Muscle mitochondrial catalase expression prevents neuromuscular junction disruption, atrophy, and weakness in a mouse model of accelerated sarcopenia. J. Cachexia Sarcopenia Muscle 12, 1582–1596. 10.1002/jcsm.12768 34559475 PMC8718066

[B221] Yalcin A. Silay K. Balik A. R. Avcioğlu G. Aydin A. S. (2018). The relationship between plasma interleukin-15 levels and sarcopenia in outpatient older people. Aging Clin. Exp. Res. 30, 783–790. 10.1007/s40520-017-0848-y 29071664

[B222] Yamada M. Kimura Y. Ishiyama D. Nishio N. Otobe Y. Tanaka T. (2019). Synergistic effect of bodyweight resistance exercise and protein supplementation on skeletal muscle in sarcopenic or dynapenic older adults. Geriatr. Gerontol. Int. 19, 429–437. 10.1111/ggi.13643 30864254

[B223] Yang A. Lv Q. Chen F. Wang Y. Liu Y. Shi W. (2020). The effect of vitamin D on sarcopenia depends on the level of physical activity in older adults. J. Cachexia Sarcopenia Muscle 11, 678–689. 10.1002/jcsm.12545 32020783 PMC7296263

[B224] Yang A. Lv Q. Han Z. Dai S. Li Y. Hao M. (2025). The effects of vitamin D on muscle strength are influenced by testosterone levels. J. Cachexia Sarcopenia Muscle 16, e13733. 10.1002/jcsm.13733 39957010 PMC11830628

[B225] Yoon H. Shaw J. L. Haigis M. C. Greka A. (2021). Lipid metabolism in sickness and in health: emerging regulators of lipotoxicity. Mol. Cell. 81, 3708–3730. 10.1016/j.molcel.2021.08.027 34547235 PMC8620413

[B226] Yuan S. Larsson S. C. (2023). Epidemiology of sarcopenia: prevalence, risk factors, and consequences. Metabolism 144, 155533. 10.1016/j.metabol.2023.155533 36907247

[B227] Zhang Y. Zou L. Chen S.-T. Bae J. H. Kim D. Y. Liu X. (2021). Effects and moderators of exercise on sarcopenic components in sarcopenic elderly: a systematic review and meta-analysis. Front. Med. (Lausanne) 8, 649748. 10.3389/fmed.2021.649748 34095166 PMC8169963

[B228] Zhang X. Li H. He M. Wang J. Wu Y. Li Y. (2022). Immune system and sarcopenia: presented relationship and future perspective. Exp. Gerontol. 164, 111823. 10.1016/j.exger.2022.111823 35504482

[B229] Zhang H. Qi G. Wang K. Yang J. Shen Y. Yang X. (2023). Oxidative stress: roles in skeletal muscle atrophy. Biochem. Pharmacol. 214, 115664. 10.1016/j.bcp.2023.115664 37331636

[B230] Zhang F. Xia Y. Su J. Quan F. Zhou H. Li Q. (2024). Neutrophil diversity and function in health and disease. Signal Transduct. Target Ther. 9, 343. 10.1038/s41392-024-02049-y 39638788 PMC11627463

[B231] Zheng G. Qiu P. Xia R. Lin H. Ye B. Tao J. (2019). Effect of aerobic exercise on inflammatory markers in healthy middle-aged and older adults: a systematic review and meta-analysis of randomized controlled trials. Front. Aging Neurosci. 11, 98. 10.3389/fnagi.2019.00098 31080412 PMC6497785

[B232] Zhou H.-H. Liao Y. Zhou X. Peng Z. Xu S. Shi S. (2024). Effects of timing and types of protein supplementation on improving muscle mass, strength, and physical performance in adults undergoing resistance training: a network meta-analysis. Int. J. Sport Nutr. Exerc Metab. 34, 54–64. 10.1123/ijsnem.2023-0118 38039960

